# Supplementary material for: The X-linked trichothiodystrophy-causing gene RNF113A links the spliceosome to cell survival upon DNA damage
Source: Nat Commun. 2020 Mar 9;11:1270. doi: 10.1038/s41467-020-15003-7 (PMC7062854; doi:10.1038/s41467-020-15003-7)
Supplement: Supplementary file 1 — Supplementary Information [file 41467_2020_15003_MOESM1_ESM.pdf]

The X-linked trichothiodystrophy-causing gene RNF113A links the spliceosome to cell survival upon DNA damage by Shostak et al.

Supplementary Figures

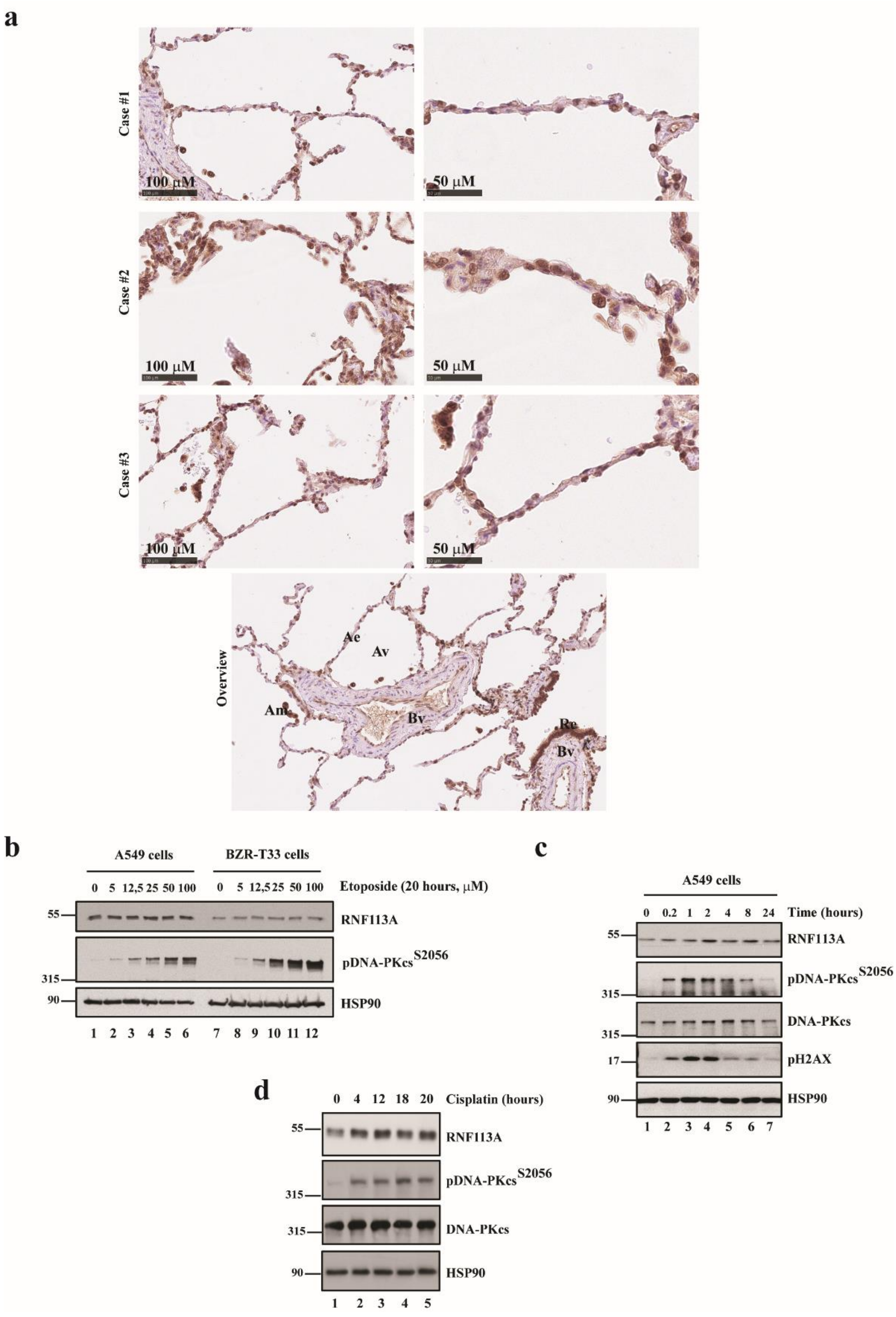

Supplementary Figure 1: γ-irradiation but not Etoposide induce RNF113A expression in lung cancer cells. a. RNF113A is weakly expressed in normal lung epithelial cells. "Ae = Alveolus; Bv = Blood Vessel, Am = Alveolar Macrophage, Ae = Alveolar Epithelium, Re =

Respiratory Epithelium. Note that there are unspecific stainings in both Re and Am (both very typical for antibodies). **b.** Etoposide fails to induce RNF113A expression in lung cancer cells. A549 or BZR-T33 cells were treated with Etoposide for 20 hours at the indicated concentrations and the resulting cell extracts were subjected to western blot (WB) analyses using the indicated antibodies. **c.** RNF113A expression is induced in γ-irradiated lung cancer cells. A549 cells were γ-irradiated (8 Gy) for the indicated periods of time and WB analyses were conducted on the resulting extracts. **d**. RNF113A expression at the protein level is induced by Cisplatin (25 μM) in normal human dermal fibroblasts.

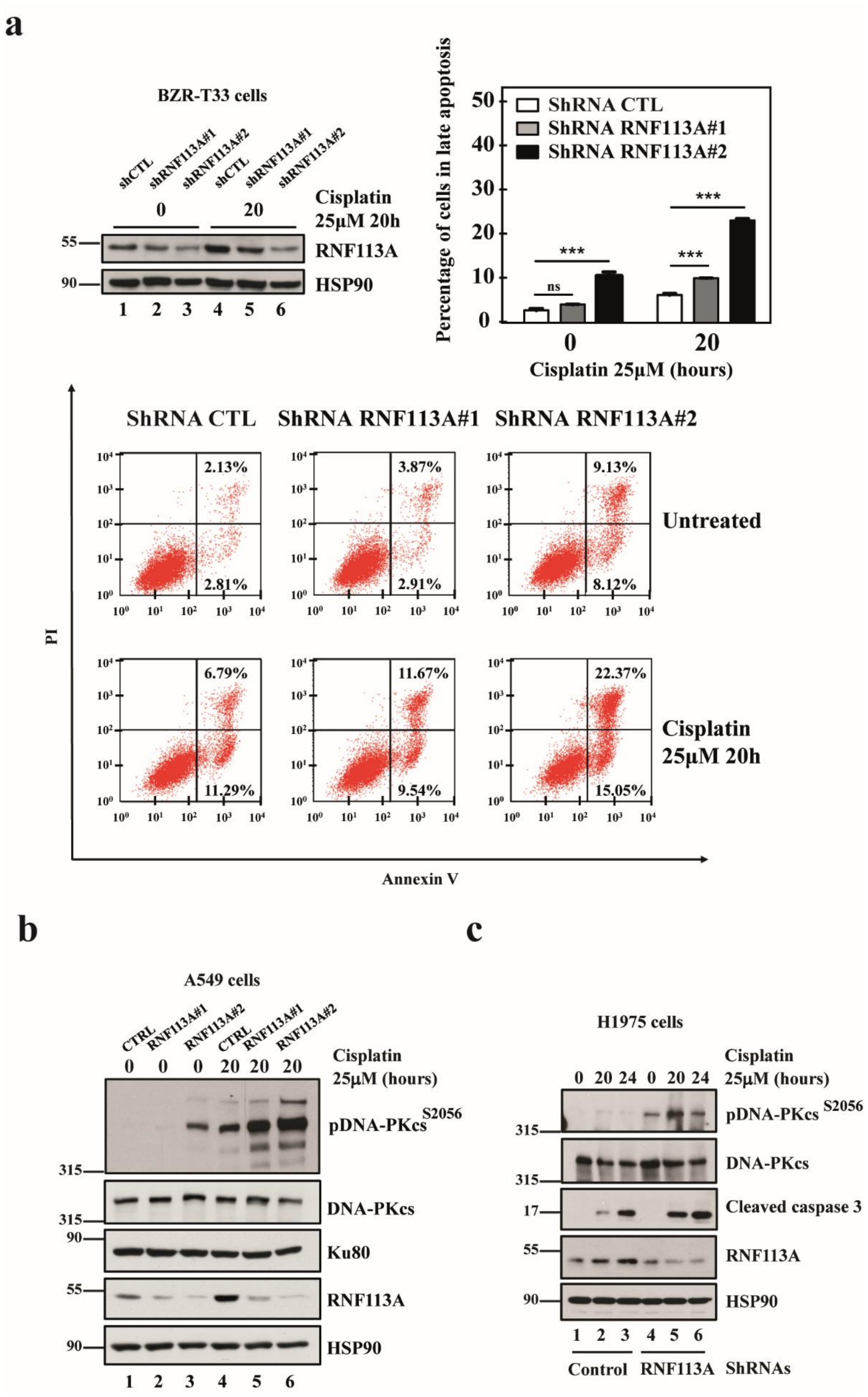

Supplementary Figure 2: RNF113A protects from Cisplatin-dependent cell death. a. RNF113A deficiency enhances cell death upon DNA damage. On the left, BZR-T33 cells were

infected with a control lentiviral construct (“ShCTRL”) or with constructs targeting two distinct sequences of the RNF113A transcript (“ShRNF113A#1 and ShRNF113A#2). The resulting cell extracts were subjected to WB analyses to assess RNF113A expression. On the right and at the bottom, cell survival upon Cisplatin treatment (25 μM for 20 hours) in control and RNF113A-depleted BZR-T33 cells was assessed by FACS. Representative FACS analyses are illustrated. The percentage of cells in early or late apoptosis is mentioned. FACS data from two independent experiments are also illustrated in the histogram (Student t-test, p-values: ***< 0.001). ns= non significant. **b.** and **c.** RNF113 deficiency enhances Cisplatin-dependent DNA-PKcs phosphorylation. Control or RNF113A-depleted A549 (b) or H1975 (c) cells were untreated or stimulated with Cisplatin (25 μM) at the indicated periods of time and the resulting cell extracts were subjected to WB analyses using the indicated antibodies.

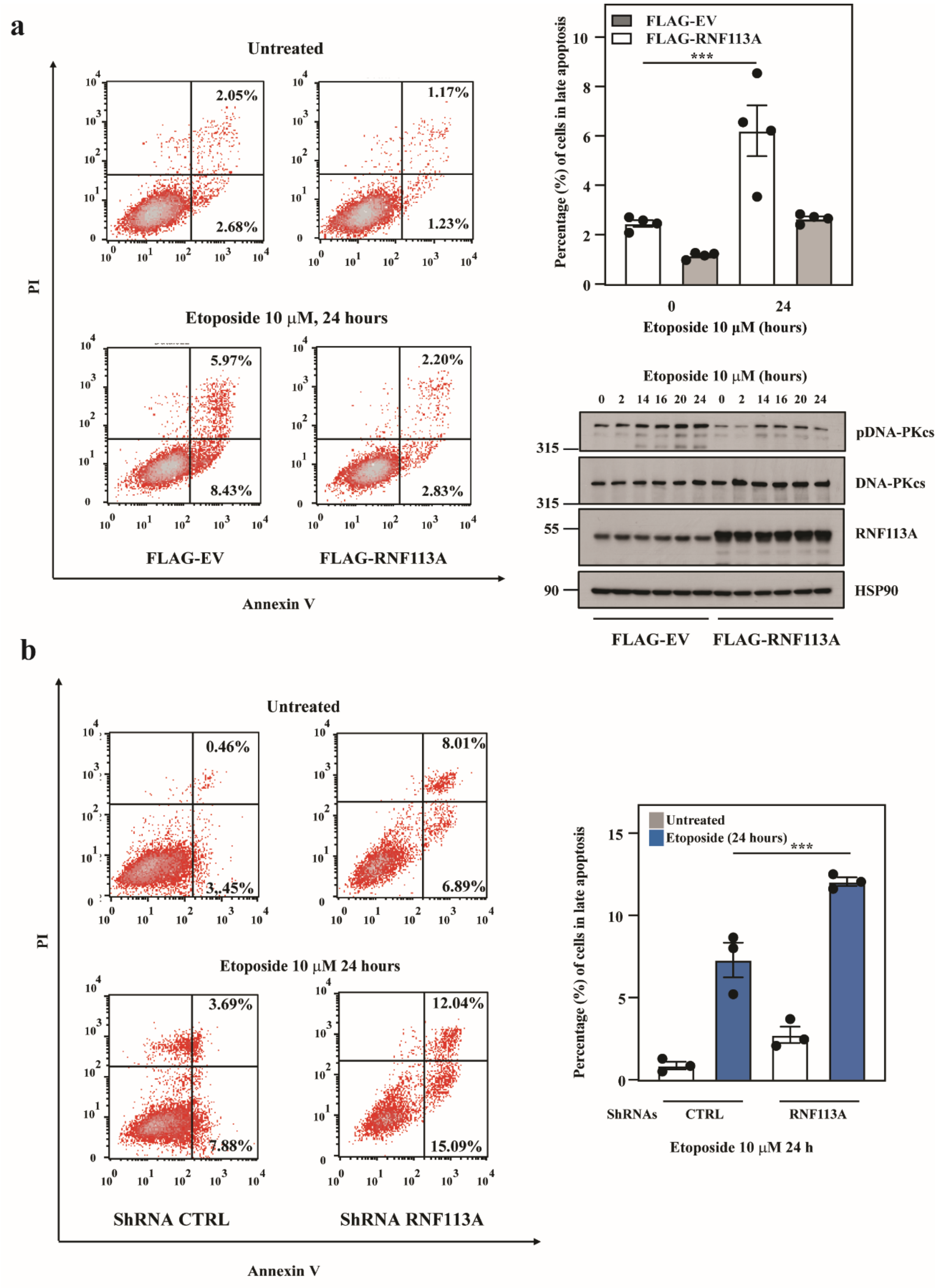

Supplementary Figure 3: RNF113A protects from Etoposide-dependent cell death. a. RNF113A overexpressed limits cell death triggered by Etoposide. Control or RNF113A-overexpressing A549 cells were treated or not with Etoposide (10 µM for 24 hours) and the

resulting cells were subjected to FACS analyses to assess cell death. Representative FACS analyses are illustrated. The percentage of cells in early or late apoptosis is mentioned. FACS data from two independent experiments are also illustrated in the histogram (Student t-test, p-values: ***< 0.001). Cell extracts were also subjected to WB analyses to assess RNF113A expression and DNA-PKcs phosphorylation. **b.** RNF113A deficiency enhances cell death upon DNA damage. A549 cells were infected with a control lentiviral construct (“ShCTRL”) or with a constructs targeting the RNF113A transcript (“ShRNF113A). On the left, cell survival upon Etoposide treatment (10 μM for 24 hours) in control and RNF113A-depleted A549 cells was assessed by FACS. Representative FACS analyses are illustrated. The percentage of cells in early or late apoptosis is mentioned. FACS data from two independent experiments are also illustrated in the histogram (Student t-test, p-values: ***< 0.001).

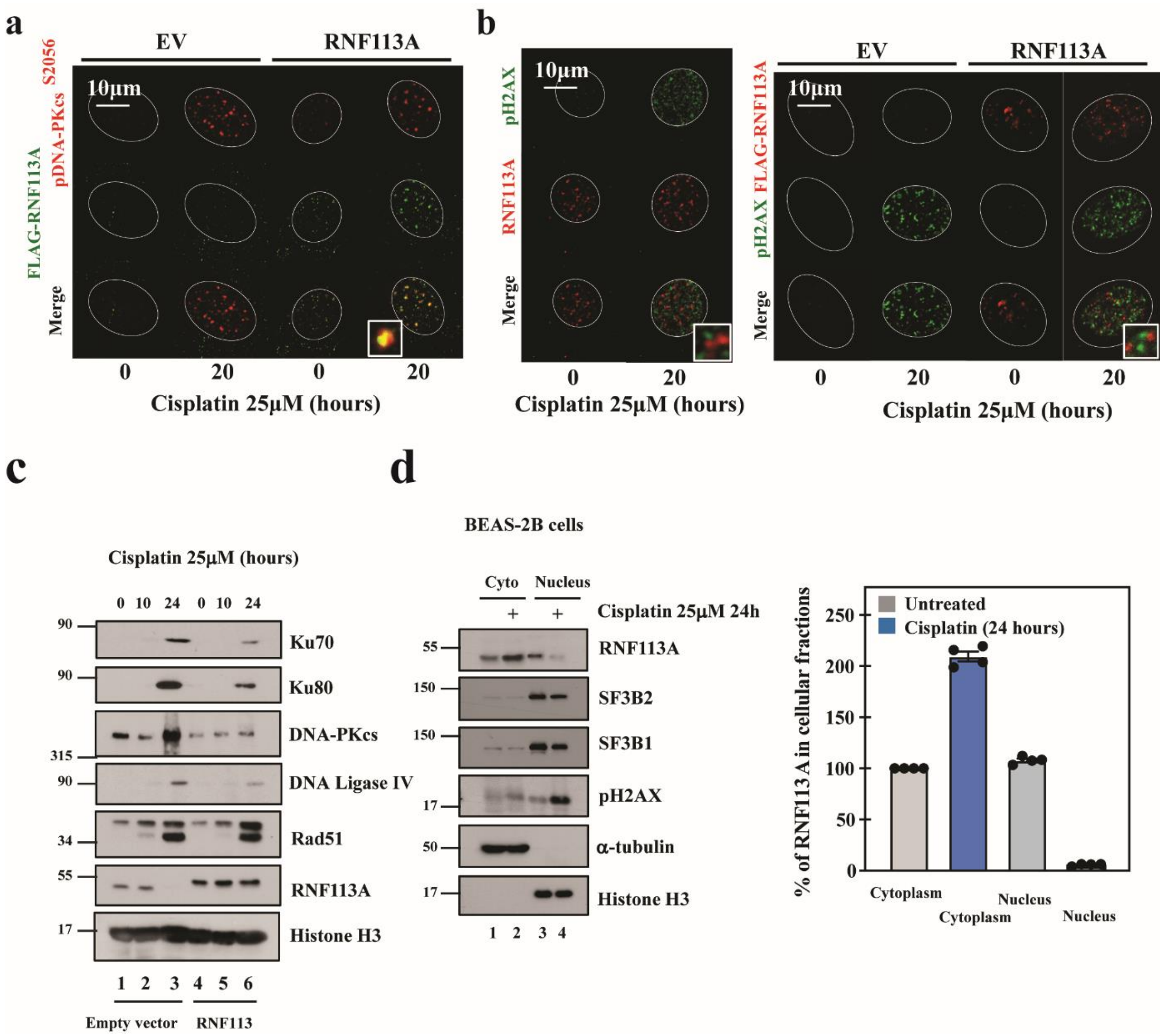

Supplementary Figure 4: RNF113A controls the recruitment of NHEJ factors on chromatin upon DNA damage and is localized both in the cytoplasm and in the nucleus. **a.** RNF113A colocalizes with DNA-PKcs S2056 foci in Cisplatin-treated cells. Control versus RNF113A-overexpressing A549 cells were stimulated with Cisplatin (25 µM) for 20 hours and immunofluorescent detections of FLAG-RNF113A and pDNA-PKcs (Serine 2056) were performed in fixed cells after pre-extraction with the CSK + RNase A buffer. Inserts represent a fivefold zoom. Nucleus are shown with a dotted line. **b.** RNF113A does not colocalize with pH_2AX foci in Cisplatin-treated cells. A549 cells or control versus RNF113A-overexpressing A549 cells (left and right panels, respectively) subjected to Cisplatin were fixed and immunofluorescence analyses of were carried out to detect RNF113A and pH_2AX after pre-

extraction with the CSK + RNase A buffer. Inserts represent a fivefold zoom. **c.** The recruitment of NHEJ factors on chromatin is regulated by RNF113A upon DNA damage. Control versus RNF113A-overexpressing A549 cells were treated or not with Cisplatin (25 µM) for 20 hours and WB analyses using the indicated antibodies were carried out on chromatin fractions after pre-extraction with the CSK + RNase A buffer. **d.** RNF113A shuttles to the cytoplasm upon DNA damage. BEAS-2B cells were treated or not with Cisplatin and the resulting nuclear and cytoplasmic extracts were subjected to WB analyses. A quantification of the pool of both cytoplasmic and nuclear RNF113A in cells subjected or not to Cisplatin treatment is provided.

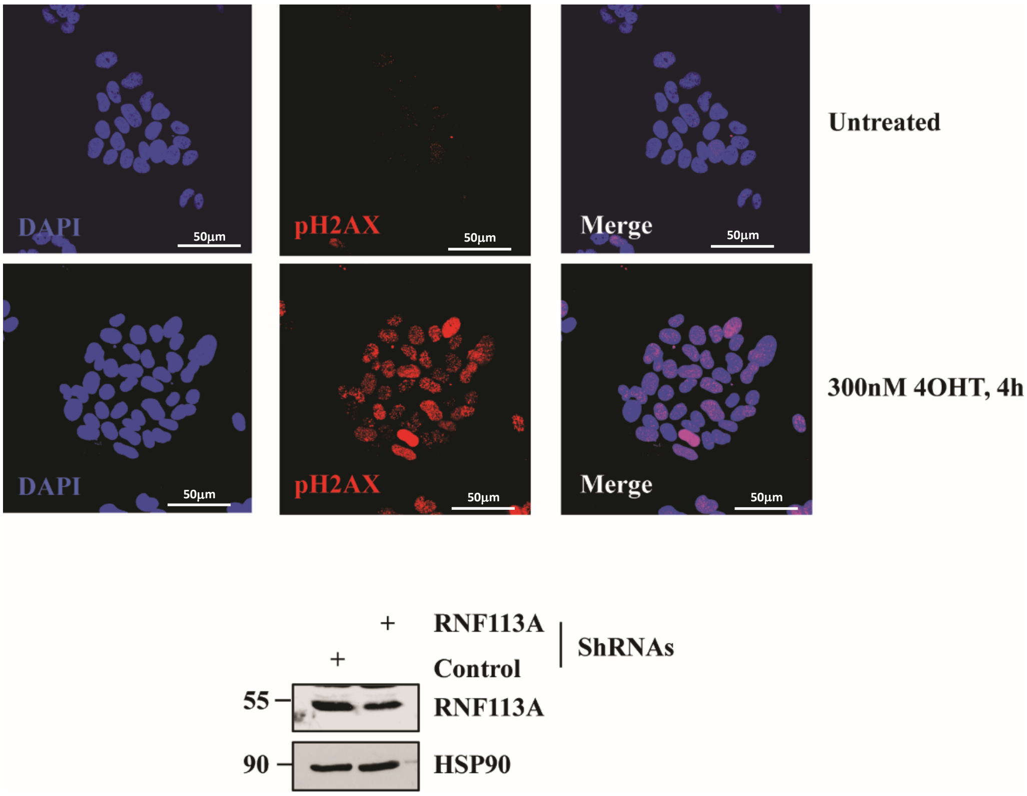

Supplementary Figure 5: DIvA U2-OS cells show signs of DNA damage upon Tamoxifen administration. On the top, immunofluoresence are illustrated to detect pH_2AX^+ cells in DIvA U2-OS cells left untreated or stimulated with 4-hydroxy Tamoxifen (4OHT) (300 nM for 4 hours). At the bottom, generation of control and RNF113A-depleted DIvA U2-OS cells using the corresponding lentiviral constructs.

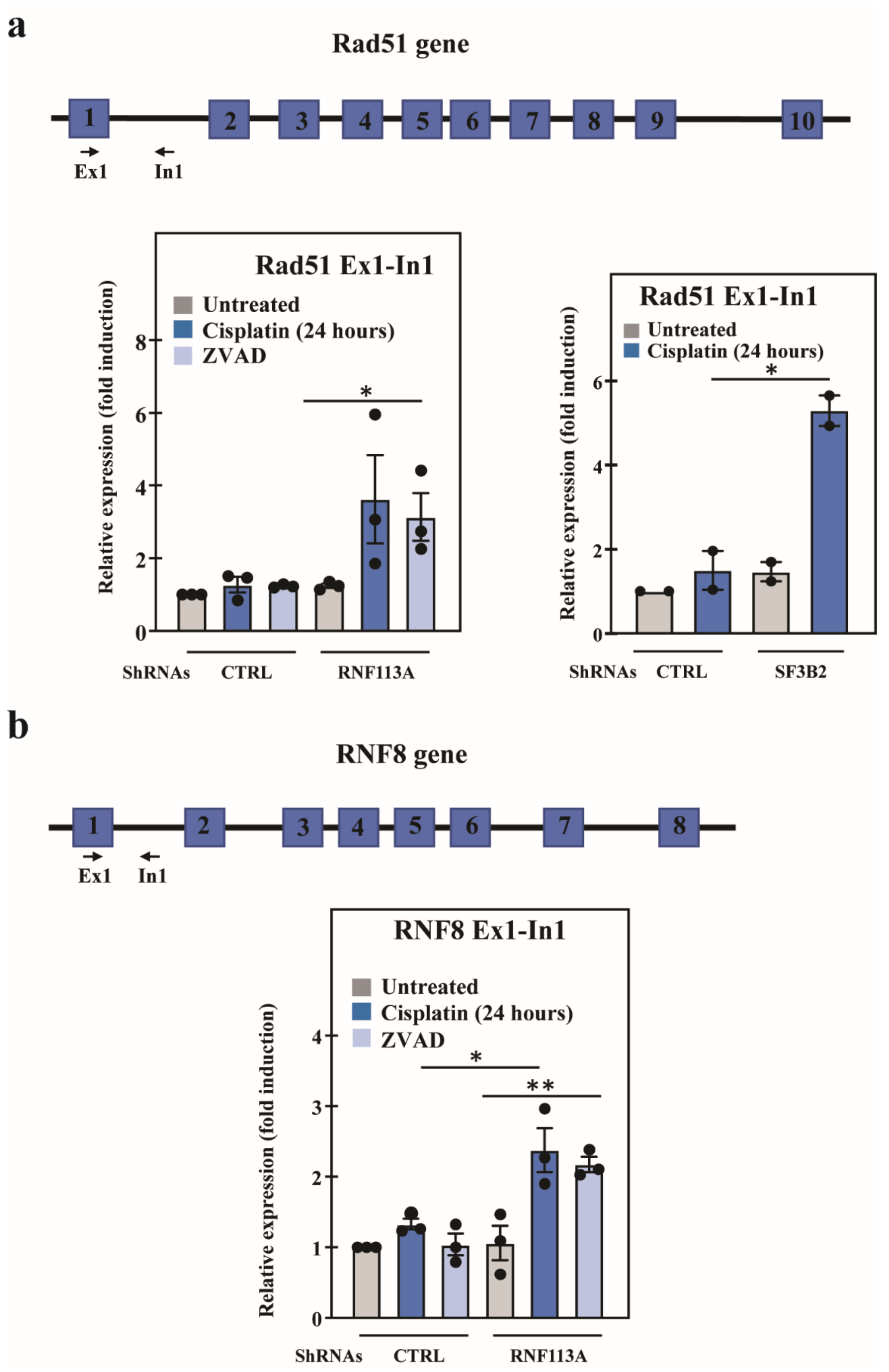

Supplementary Figure 6: RNF113A controls the splicing of both Rad51 and RNF8 in lung cancer cells. RNF113A deficiency interferes with the splicing of *RAD51* and *RNF8* (a and b, respectively). A schematic representation of both *RAD51* and *RNF8* genes is illustrated. Primers

used in these experiments are also depicted. Real-Time PCR analyses were conducted to quantify Rad51 or RNF8 pre-mRNAs (in which intron 1 is retained) in control, RNF113A- or SF3B2-depleted A549 cells treated or not with Cisplatin (25 μM for 24 hours) (left and right panel, respectively). Rad51 or RNF8 pre-mRNAs levels in untreated control cells were set to 1 and levels in all other experimental conditions were relative to that after normalization with β-actin. Data from two Real-time PCR independent analyses performed in triplicates (means ± SD) are shown (***=p<0.001, **= p<0.01, *= p<0.05, Student t-test).

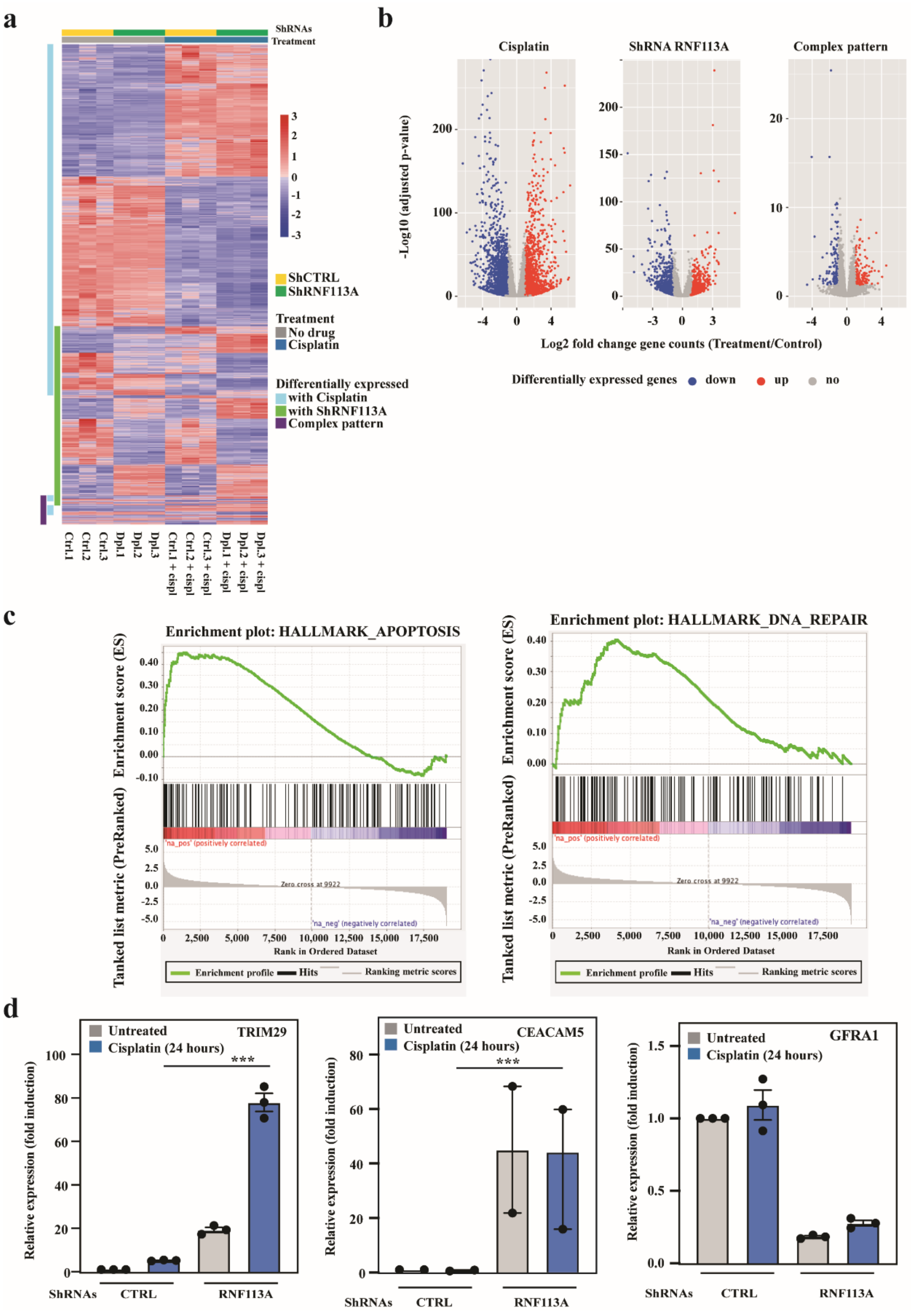

Supplementary Figure 7: RNF113A regulates the expression of multiple genes in lung cancer cells. a.. and **b.** Results of the differential expression analysis performed at the gene

level on RNAseq data for control and RNF113A-depleted A549 cells treated or not with Cisplatin. (**a**) Heatmap showing for all replicates relative gene expression levels for all significantly differentially expressed genes. For each gene, relative expression measurements are expressed in the form of Z-scores of the rlog-transformed normalized counts from DESeq2. (**b**) Volcano plots showing significance as a function of the expression fold change for multifactor models evaluating the changes induced (i) in Cisplatin treated versus untreated cells when accounting for the potential effect of RNF113A depletion (left), (ii) in RNF113A-depleted versus control cells when accounting for the potential effect of Cisplatin (middle), and (iii) upon RNF113A depletion but depending on the Cisplatin-treatment status (right). Significance threshold: 5% FDR q-value. Additional threshold on effect size: two-fold change. For “shRNF113A versus shCTRL” and for “Cisplatin versus no drug”, log2 fold changes used in the plots are corrected for the over-dispersion due to low counts using DESeq2 shrinkage procedure. For the complex pattern, the fold change is not shrunk and calculated as follow: (shRNF113A + Cisplatin / shRNF113A – no drug) / (shCTRL + Cisplatin / shCtrl – no drug). **c.** Enrichment of genes linked to apoptosis and DNA repair in Cisplatin versus untreated A549 cells, as evidenced by Gene Set Enrichment analyses (GSEA). **d.** Increased *TRIM29* and *CEACAM5* but decreased *GFRA1* expression upon RNF113A deficiency in both untreated and Cisplatin-stimulated A549 cells. mRNA levels in untreated control cells are set to 1 and levels in other experimental conditions are relative to that after normalization with β-actin. Data from two Real-time PCR independent analyses performed in triplicates (means $\pm$ SD) are shown.

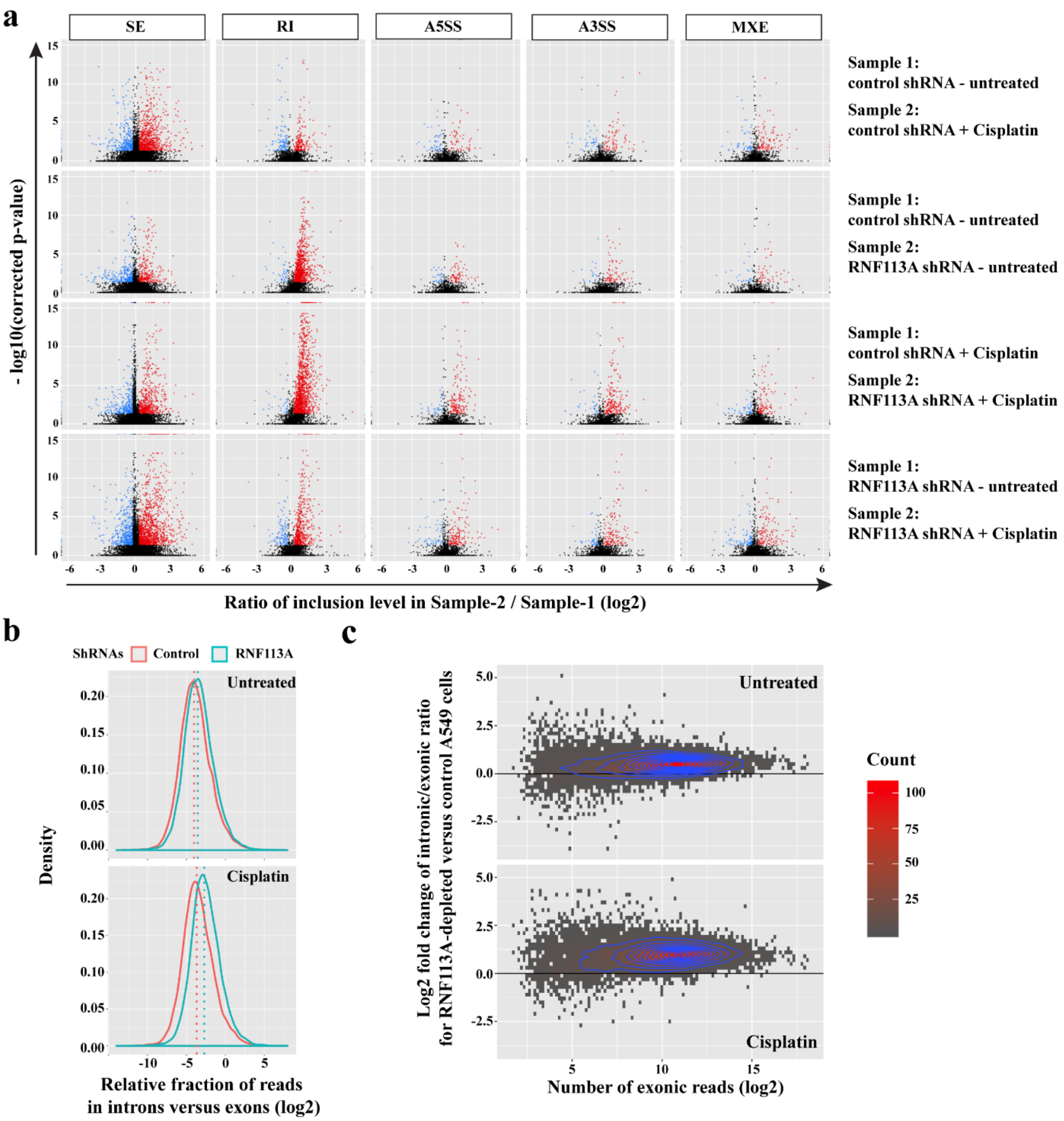

Supplementary Figure 8: Increased intron retention in mRNAs upon RNF113A deficiency, especially in Cisplatin-treated lung cancer cells. a. Analysis of alternative splicing (AS) at the level of individual splicing events with rMATS. Volcano plots comparing inclusion level of different AS events between RNF113A-depleted and control A349 cells treated or not with Cisplatin are illustrated. Blue dots represent events with higher inclusion level in sample 1 and red dots with higher inclusion level in sample 2. Significant events are defined as FDR<5% and delta inclusion level (|$\Delta\Psi$|) of at least 20%. **b.-c.** Analysis of intron

retention at the level of each protein-coding gene. **b.** Distribution of the gene ratio of mRNAseq reads in intronic versus exonic regions (average of three replicates) for RNF113A-depleted and control A549 cells treated or not with Cisplatin. All pairwise comparisons significant (p-value $< 2.2\ 10^{-16}$) by Wilcoxon signed rank test (paired tests). Dotted line = median for each condition. **c.** Density plot of the log2 fold change of the gene intronic/exonic read ratio between RNF113A-depleted and control A549 cells treated or not with Cisplatin as a function of the number of exonic reads (in log2) is illustrated. Although increased dispersion is observed, as expected, for genes with lower gene counts, there is a striking global increase of the intronic/exonic read ratio upon RNF113A depletion, especially in Cisplatin-treated A549 cells.

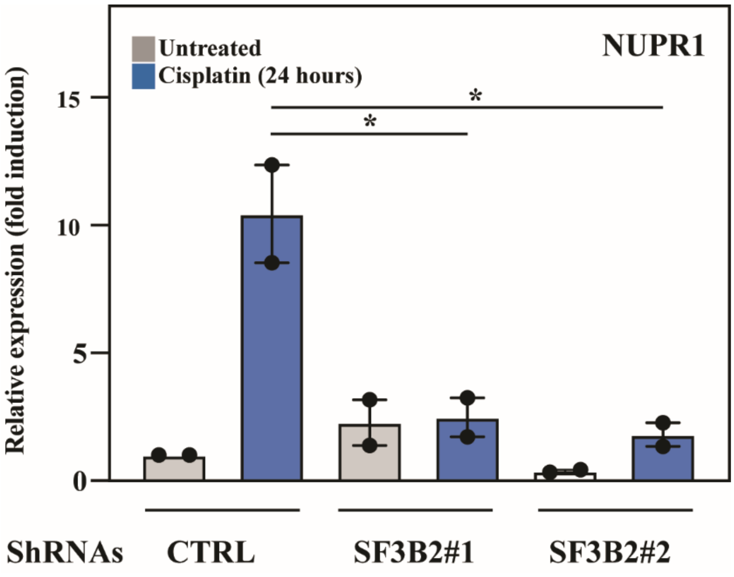

Supplementary Figure 9: SF3B2 deficiency interferes with the production of the NUPR1-encoding mRNA. Real-Time PCRs were carried out with extracts from control versus SF3B2-depleted A549 cells treated or not with Cisplatin (25 μM for 24 hours) and mRNA levels of the NUPR1-encoding transcript were quantified in all experimental conditions. NUPR1 mRNA levels in unstimulated cells is set to 1 and levels in other experimental conditions are relative to that after normalization with β-actin. Data from two Real-time PCR independent analyses performed in triplicates (means ± SD) (***=p<0.001, Student t-test).

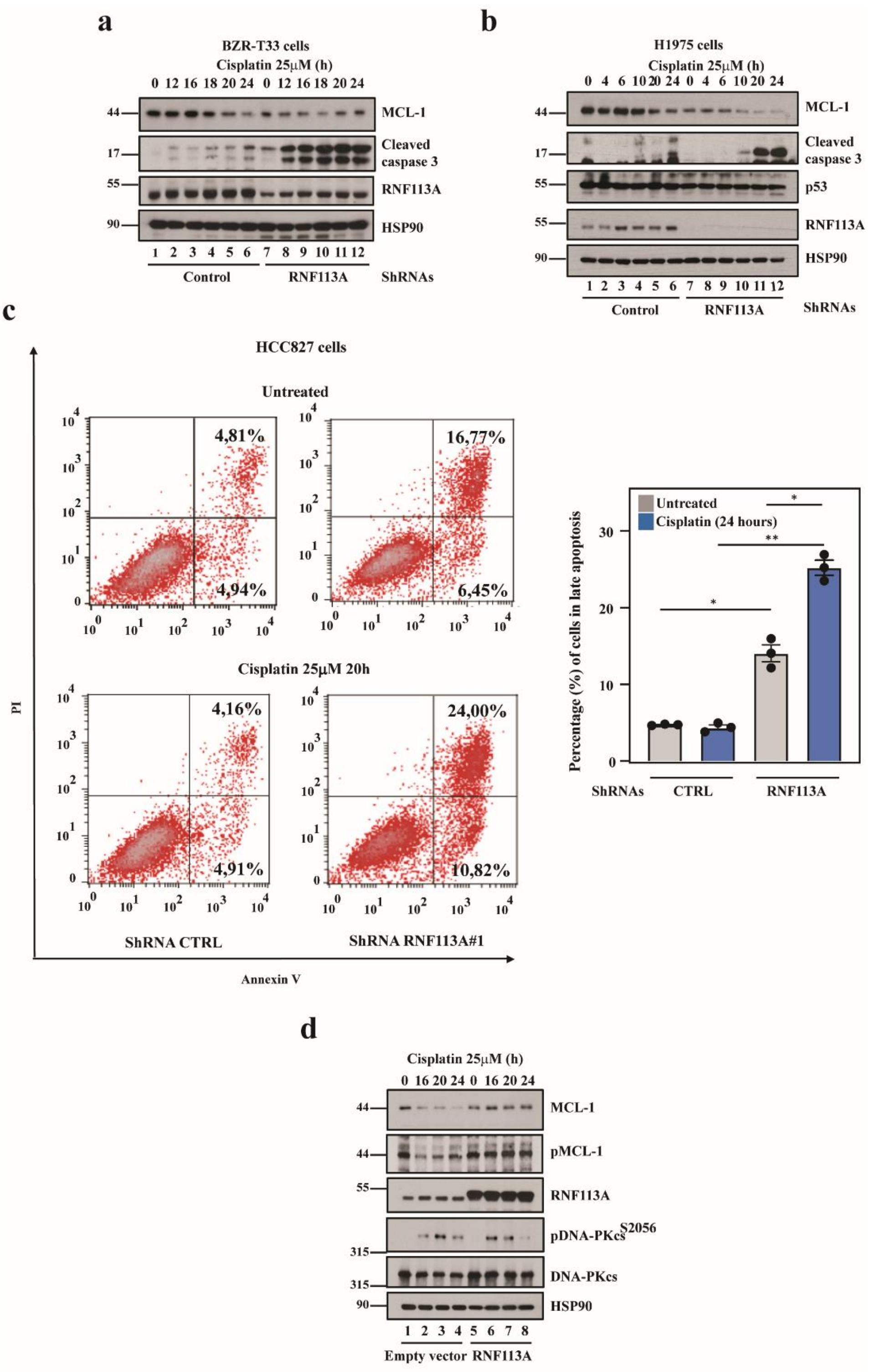

Supplementary Figure 10: RNF113A promotes MCL-1 stability in lung cancer cells. a. and **b.** RNF113A controls MCL-1 protein levels in Cisplatin-treated lung cancer cells. Control

or RNF113A-depleted BZR-T33 (a) or H1975 (b) cells were treated or not with Cisplatin (25 μM) for the indicated periods of time and the resulting cell extracts were subjected to WB analyses. **c.** RNF113A deficiency enhances cell death upon Cisplatin treatment in Caspase 3-negative lung cancer cells. HCC827 cells were infected with a control lentiviral construct ("ShCTRL") or with a construct targeting the RNF113A transcript ("ShRNF113A#1") and cell survival in unstimulated cells or upon Cisplatin treatment (25 μM for 20 hours) was assessed by FACS (left panels). The percentage of cells in early or late apoptosis is mentioned. On the right, FACS data from two independent experiments are also illustrated in the histogram (Student t-test, p-values: ***< 0.001). **d.** RNF113A overexpression enhances MCL-1 levels upon DNA damage in lung cancer cells. Control or RNF113A-overexpressing A549 cells were treated or not with Cisplatin (25 μM) at the indicated periods of time and the resulting cell extracts were subjected to WB analyses using the indicated antibodies.

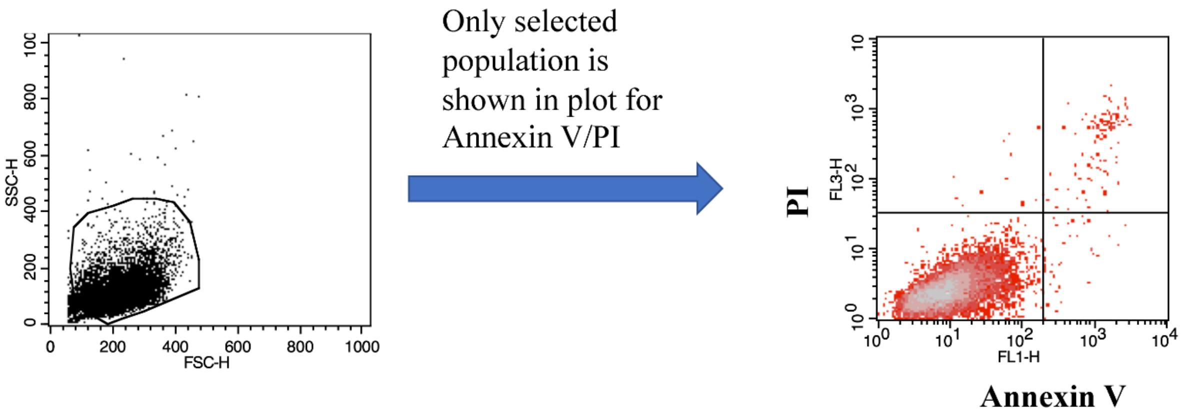

Supplementary Figure 11: Gating strategy used for FACS analyses. The gating strategy used was forward and side scatter gating to remove debris and other events of non-interest while preserving cells based on size and or complexity.

Antibody	Company	Catalogue number	Dilution
pH_2AX (S139)	Cell Signaling Technology	9718	1/1000
IgG	Cell Signaling Technology	2729	Same as antibody of interest
Anti-gamma pH2AX (S139)	Abcam	ab2893	1/500
HSP90	Santa Cruz Biotechnology	Sc-13119	1/1000
DNA-PKcs	Abcam	Ab1832	1/500
pDNA-PKcs (S2056)	Abcam	Ab18192	1/500
pChk1 (S345)	Cell Signaling Technology	2348	1/1000
Chk1	Cell Signaling Technology	2360	1/1000
Ku70	Santa Cruz Biotechnology	Sc-9033	1/1000
Ku80	Abcam	Ab3107 and Ab80592	1/500
RNF113A	Sigma	HPA000160	1/2000
Rabbit RNF113A	Phoenix Pharmaceutical	Made for this study	1/2000
pP53S15	Cell Signaling Technology	9286S	1/1000
FLAG	Sigma	F3166	1/5000
c-Myc	Santa Cruz Biotechnology	Sc-789 and Sc-40	1/1000
DNA-RNA Hybrids S9.6	Kerafast	ENH001	1/200
SF3B1	Cell Signaling Technology	14434S	1/1000
SF3B2	Bethyl Laboratories	A301-605A	1/1000
RNF8	Santa Cruz Biotechnology	Sc-271462	1/500
Rad51	Abcam	Ab213	1/500
MCL-1	Santa Cruz Biotechnology	Sc-819	1/1000
pMCL-1	Cell Signaling Technology	4579S	1/1000
C/EBPβ	Santa Cruz Biotechnology	Sc-7962	1/1000
P53	Santa Cruz Biotechnology	Sc-6243	1/1000
MDM2	Santa Cruz Biotechnology	Sc-965	1/500
Cleaved caspase 3	Cell Signaling Technology	9661S	1/1000
Caspase 3	Cell Signaling Technology	9662S	1/1000
SAT1	Cell Signaling Technology	61586S	1/1000
Noxa1	LSBio	LS-C313065	1/1000
β-Actin	Cell Signaling Technology	4967S	1/1000

pERK1/2 (Y202/T204)	Cell Signaling Technology	9101	1/1000
E-cadherin	BD Biosciences	610182	1/1000
Lamin A/C	Santa Cruz Biotechnology	Sc-7293	1/1000
Histone H3	Abcam	Ab1791	1/5000
Caspase 8	Cell Signaling Technology	9746S	1/1000
Caspase 9	Cell Signaling Technology	7237S	1/1000
RPL7	Bethyl Laboratories	A300-741A	1/1000
DNA Ligase IV	Abcam	Ab26039	1/1000
USP9X	Bethyl Laboratories	A301-350A	1/1000
Ubiquitin remnant K-ε-GG antibody	Cell Signaling Technology	5562	1/1000
Goat secondary antibodies (anti-rabbit)	GE Healthcare UK Limited	NA934V	1/5000
Goat secondary antibodies (anti-mouse)	GE Healthcare UK Limited	NA931V	1/5000
Alexa Fluor 488 goat anti-mouse IgG(H+L)	Life Technologies	A11001	1/500
Alexa Fluor 568 goat anti-rabbit IgG(H+L)	Life Technologies	A11011	1/500

Supplementary Table 1: List of antibodies used in this study.

**Oligonucleotides**		
Noxa1 for	IDT DNA	CACTTGGAGCCCGTGGATTT
Noxa1 rev	IDT DNA	ATCATGGTTCGCTCCTGGTC
Noxa1 exon7-9 for	IDT DNA	GGACCAGGAGCGAACCAT
Noxa1 exon7-9 rev	IDT DNA	AGTGCCCGCAGGCTGGACAG
TRIM29 for	IDT DNA	GCTCTTCTGCCAGACCGAC
TRIM29 rev	IDT DNA	CTTTTGCAATGACAGCTCCGT
CEACAM5 for	IDT DNA	GGACCACAGTCACGACGA
CEACAM5 rev	IDT DNA	CCTCATCCTCCACGGGGT
Nupr1 for	IDT DNA	CCTCTATAGCCTGGCCCATT
Nupr1 rev	IDT DNA	GCAGGAGTCAGAGGTGAAGT
GFRA1 for	IDT DNA	CAGCAAGTGGAGCACATTCC
GFRA1 rev	IDT DNA	ACGCCGACCTGTACTTCTTG
SAT1 for	IDT DNA	TTGCAGAAGTGCCGAAAGAGC
SAT1 rev	IDT DNA	AGAAGTGCATGCTGCTGCAGCG
Nupr1 alt for	IDT DNA	CCGGAGGACGAGGACTCCAG
Nupr1 alt rev	IDT DNA	AGCTCTGTCTCAGCGCCGTGC
RNF113a for	IDT DNA	GTTCGACCGGAAAAGAAGCG
RNF113a rev	IDT DNA	TCCTCTTCGCTGCTCAAGTC
CHIP site1 for	IDT DNA	CCTCTACTTGCTTCCGGTTG
CHIP site1 rev	IDT DNA	AGGTTGCAGTGAGCGATTCT
CHIP site2 for	IDT DNA	AGCGAAACTCCGTCTCAAAA
CHIP site2 rev	IDT DNA	GGATCAACCGAGGTCAGAAG
CHIP site3 for	IDT DNA	CTCCCAAAGTGCTGGGATTA
CHIP site3 rev	IDT DNA	GGGGAAAATGAATCCTGATG
CHIP site4 for	IDT DNA	AAAGTCACAGGTACCAACTTAATCC
CHIP site4 rev	IDT DNA	GGGACATTCCAGAGAAATGG

Supplementary Table 2: List of primers used in this study.
