# Supplementary material for: The X-linked trichothiodystrophy-causing gene RNF113A links the spliceosome to cell survival upon DNA damage
Source: Nat Commun. 2020 Mar 9;11:1270. doi: 10.1038/s41467-020-15003-7 (PMC7062854; doi:10.1038/s41467-020-15003-7)
Supplement: Supplementary file 3 — Description of Additional Supplementary Files [file 41467_2020_15003_MOESM3_ESM.pdf]

Description of Additional Supplementary Files

File Name: Supplementary Data 1
Description: **List of clinical cases of lung cancer tested for RNF113A expression.**

File Name: Supplementary Data 2
Description: **Differential expression analysis results for genes in control or RNF113A-depleted A549 cells treated or not with Cisplatin.** Table with DESeq2 analysis results and containing, for each gene, the average of the normalized expression level across all samples, the log2 of the expression fold change for three different comparisons (see Methods) and the adjusted p-value for each of these comparisons. Log2 fold change for "shRNF113A versus shCtrl" and for "Cisplatin versus no drug" are corrected for the over-dispersion due to low counts using DESeq2 shrinkage procedure. "Complex patterns" refers to the model including an interaction term. In this column, the log2 fold change is not shrunk and corresponds to the ratio of ratio for the two factors: (shRNF113A + Cisplatin / shRNF113A – no drug) / (shCtrl + Cisplatin / shCtrl – no drug).

File Name: Supplementary Data 3-7
Description: **Alternative splicing analysis at the level of individual splicing events and per category of event. Tables presenting the results of the rMATS analysis (one table per category of event).** Categories are as follow: SE = skipped exons, MXE = mutually exclusive exons, RI = retained intron, A5SS = alternative 5' splicing site and A3SS = alternative 3' splicing site. For each type of event, the first three columns (A-C) indicate the two types of samples compared (S1 and S2) and are followed by the gene identifiers (Ensembl Gene ID and Gene symbol). The next four columns (F-I) provide the mean inclusion level in the first (S1) and in the second (S2) type of samples, their ratio and FDR (False Discovery Rate). They are followed by columns providing information about the event itself (genomic locations in hg19). Samples are as follow: C = control shRNA – no drug, Ccis = control shRNA + Cisplatin, D = RNF113A shRNA – no drug, Dcis = RNF113A shRNA + Cisplatin.

File Name: Supplementary Data 8
Description: **Intron retention analysis at the gene level.** Table providing the results of the global intron retention analysis at the gene level. For each gene, the table provides the mean exon counts across all samples as well as the mean and the standard deviation of the ratio between the number of reads in introns over the number of reads in exons for all four conditions.
