# Supplementary material for: The X-linked trichothiodystrophy-causing gene RNF113A links the spliceosome to cell survival upon DNA damage
Source: Nat Commun. 2020 Mar 9;11:1270. doi: 10.1038/s41467-020-15003-7 (PMC7062854; doi:10.1038/s41467-020-15003-7)
Supplement: Supplementary file 12 — Reporting Summary [file 41467_2020_15003_MOESM12_ESM.pdf]

nature research

Corresponding author(s): alain.chariot@uliege.be

Reporting Summary

Nature Research wishes to improve the reproducibility of the work that we publish. This form provides structure for consistency and transparency in reporting. For further information on Nature Research policies, see Authors & Referees and the Editorial Policy Checklist.

Statistical parameters

When statistical analyses are reported, confirm that the following items are present in the relevant location (e.g. figure legend, table legend, main text, or Methods section).

n/a	Confirmed	
☐	☒	The exact sample size (*n*) for each experimental group/condition, given as a discrete number and unit of measurement
☐	☒	An indication of whether measurements were taken from distinct samples or whether the same sample was measured repeatedly
☐	☒	The statistical test(s) used AND whether they are one- or two-sided<br>*Only common tests should be described solely by name; describe more complex techniques in the Methods section.*
☐	☒	A description of all covariates tested
☐	☒	A description of any assumptions or corrections, such as tests of normality and adjustment for multiple comparisons
☐	☒	A full description of the statistics including central tendency (e.g. means) or other basic estimates (e.g. regression coefficient) AND variation (e.g. standard deviation) or associated estimates of uncertainty (e.g. confidence intervals)
☐	☒	For null hypothesis testing, the test statistic (e.g. *F*, *t*, *r*) with confidence intervals, effect sizes, degrees of freedom and *P* value noted<br>*Give P values as exact values whenever suitable.*
☐	☒	For Bayesian analysis, information on the choice of priors and Markov chain Monte Carlo settings
☐	☒	For hierarchical and complex designs, identification of the appropriate level for tests and full reporting of outcomes
☐	☒	Estimates of effect sizes (e.g. Cohen's *d*, Pearson's *r*), indicating how they were calculated
☐	☒	Clearly defined error bars<br>*State explicitly what error bars represent (e.g. SD, SE, CI)*

Our web collection on statistics for biologists may be useful.

Software and code

Policy information about availability of computer code

Data collection	DeSeq2 and rMATS were used to generate both RNASeq and splicing analyses, respectively.
Data analysis	Gene Set Enrichment Analysis (GSEA), Excell, GraphPad, Prism 8, Image J, DeSeq2, IGV 2.3.91, FlowJo LLC, rMATS.

For manuscripts utilizing custom algorithms or software that are central to the research but not yet described in published literature, software must be made available to editors/reviewers upon request. We strongly encourage code deposition in a community repository (e.g. GitHub). See the Nature Research guidelines for submitting code & software for further information.

Data

Policy information about availability of data

All manuscripts must include a data availability statement. This statement should provide the following information, where applicable:

- Accession codes, unique identifiers, or web links for publicly available datasets
- A list of figures that have associated raw data
- A description of any restrictions on data availability

• Raw data and results of the RNAseq analysis are available on the NCBI GEO website under the GSE133029 accession number:

https://www.ncbi.nlm.nih.gov/geo/query/acc.cgi?acc=GSE133029

Field-specific reporting

Please select the best fit for your research. If you are not sure, read the appropriate sections before making your selection.

☒ Life sciences ☐ Behavioural & social sciences

For a reference copy of the document with all sections, see nature.com/authors/policies/ReportingSummary-flat.pdf

Life sciences

Study design

All studies must disclose on these points even when the disclosure is negative.

Sample size	Sample size for xenograft experiment was defined by using 2-Sample T-Test method.
Data exclusions	No animal was excluded from the experiments.
Replication	For all experiments, all replicates are mentioned in the figure legends.
Randomization	No randomization of mice was used. Mice analysed were litter mates and age-matched whenever possible.
Blinding	No blinding was done during the experiment as mice were injected with control or RNF113A-depleted cells in each flanck.

Materials & experimental systems

Policy information about availability of materials

n/a	Involved in the study
☒	☐ Unique materials
☐	☒ Antibodies
☐	☒ Eukaryotic cell lines
☐	☒ Research animals
☐	☒ Human research participants

Antibodies

Antibodies used	All antibodies used in the study are listed in Table S9.
Validation	For commercial antibodies, no additional validation was performed. For our home-made anti-RNF113A antibody, the validation was performed by carrying out western blot analyses with control and RNF113A-depleted A549 cells (lentiviral infections).

Eukaryotic cell lines

Policy information about cell lines

Cell line source(s)	Cell lines source is stated in the Method section of the manuscript.
Authentication	No authentication of the cell lines was performed by us. This authentification was carried out by ATCC.
Mycoplasma contamination	Mycoplasma was checked routinely. Only cells found negative for mycoplasma were used in this study.
Commonly misidentified lines (See ICLAC register)	No commonly misidentified lines were used.

Research animals

Policy information about studies involving animals; ARRIVE guidelines recommended for reporting animal research

Animals/animal-derived materials	Information about the animals are reported in the Method section of the manuscript.<br>For xenograft experiments: 8 weeks NOD-SCID males were used.

Human research participants

Policy information about studies involving human research participants

Population characteristics	Patients from the University of Cologne were suffering from lung adenocarcinomas. A written informed consent was received from participants at the University Hospital of Cologne prior to inclusion in the study.

Method-specific reporting

n/a	Involved in the study	
☒	☐	ChIP-seq
☐	☒	Flow cytometry
☒	☐	Magnetic resonance imaging

Flow Cytometry

Plots

Confirm that:

☒ The axis labels state the marker and fluorochrome used (e.g. CD4-FITC).

☒ The axis scales are clearly visible. Include numbers along axes only for bottom left plot of group (a 'group' is an analysis of identical markers).

☒ All plots are contour plots with outliers or pseudocolor plots.

☒ A numerical value for number of cells or percentage (with statistics) is provided.

Methodology

Sample preparation	The Annexin-V-FLUOS Staining Kit (Roche) was used to access cell death following the manufacturer's instructions, using the FACSCalibur flow cytometer (BD Biosciences). For the measurement of lipid peroxidation, control or RNF113A-depleted A549 cells treated or not with Cisplatin (25 micromolar for 24 hours), were incubated for 30 minutes at 37°C in a medium supplemented with 5 micromolar C-11 BODIPY (Invitrogen). Cells were analysed on FACS Canto II and the data were generated using the FlowJo program. For ROS measurement, control or RNF113A-depleted A549 cells treated or not with Cisplatin 25 micromolar for 24 hours) were incubated for 10 minutes at 37°C in a carboxy-H2DFFDA-containing solution at a concentration of 24 micromolar (Invitrogen). Cells were analysed on FACS Canto II and the data were generated using the FlowJo program.
Instrument	For cell death assay: FACS Calibur2Lasers and FACS Canto II.
Software	FlowJo, BD FACSDiva software.
Cell population abundance	No sorting was performed.
Gating strategy	For all FACS analyses, the gating strategy used was forward and side scatter gating — to remove debris and other events of non-interest while preserving cells based on size and or complexity.

☒ Tick this box to confirm that a figure exemplifying the gating strategy is provided in the Supplementary Information.
