# Supplementary material for: The X-linked trichothiodystrophy-causing gene RNF113A links the spliceosome to cell survival upon DNA damage
Source: Nat Commun. 2020 Mar 9;11:1270. doi: 10.1038/s41467-020-15003-7 (PMC7062854; doi:10.1038/s41467-020-15003-7)
Supplement: Supplementary file 13 — Source Data [file 41467_2020_15003_MOESM13_ESM.zip › Source Data - Uncropped Gels.pptx]

## Slide 1
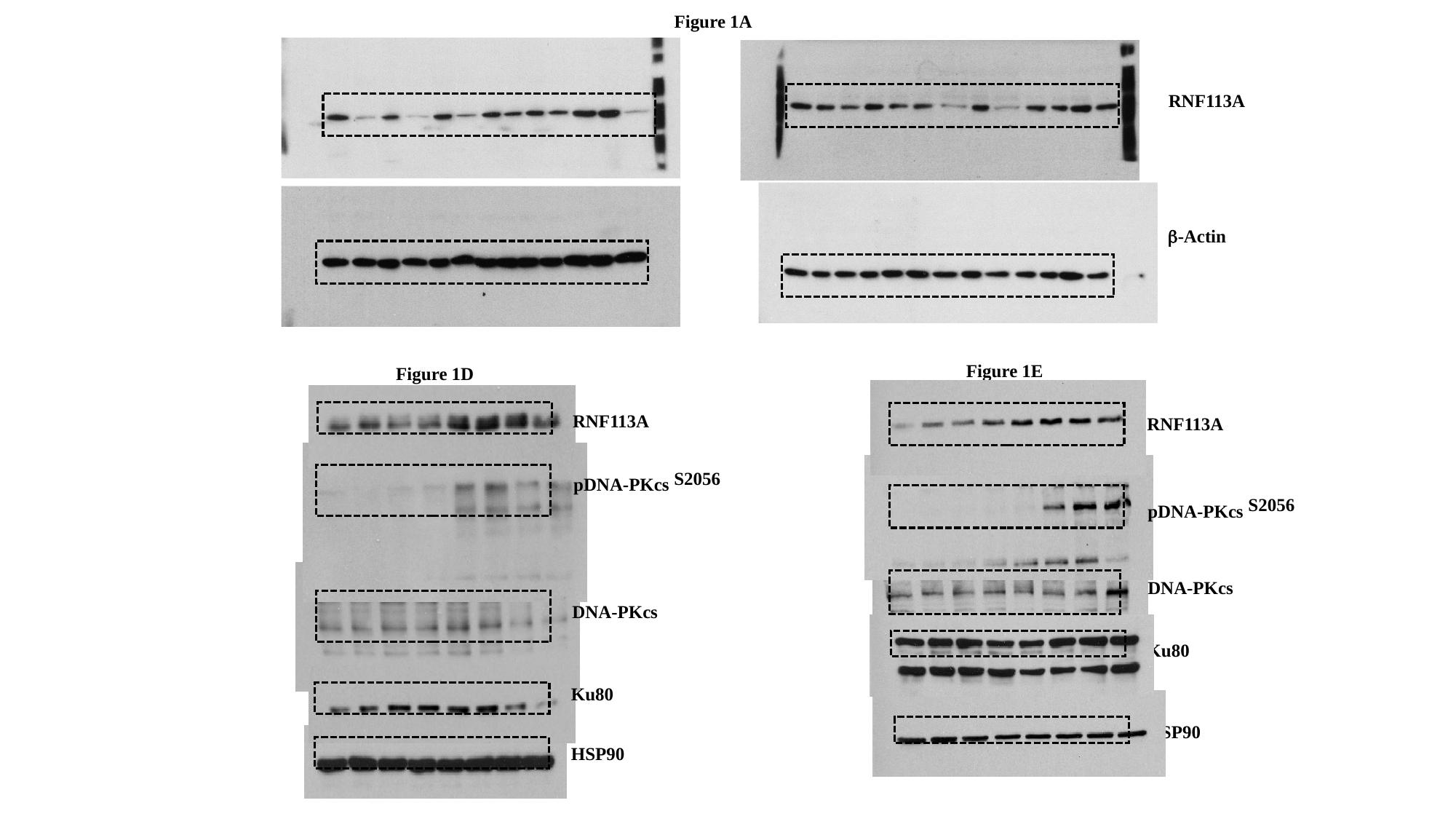

Figure 1A
RNF113A
b-Actin
Figure 1E
Figure 1D
RNF113A
RNF113A
S2056
pDNA-PKcs
S2056
pDNA-PKcs
DNA-PKcs
DNA-PKcs
Ku80
Ku80
HSP90
HSP90

## Slide 2
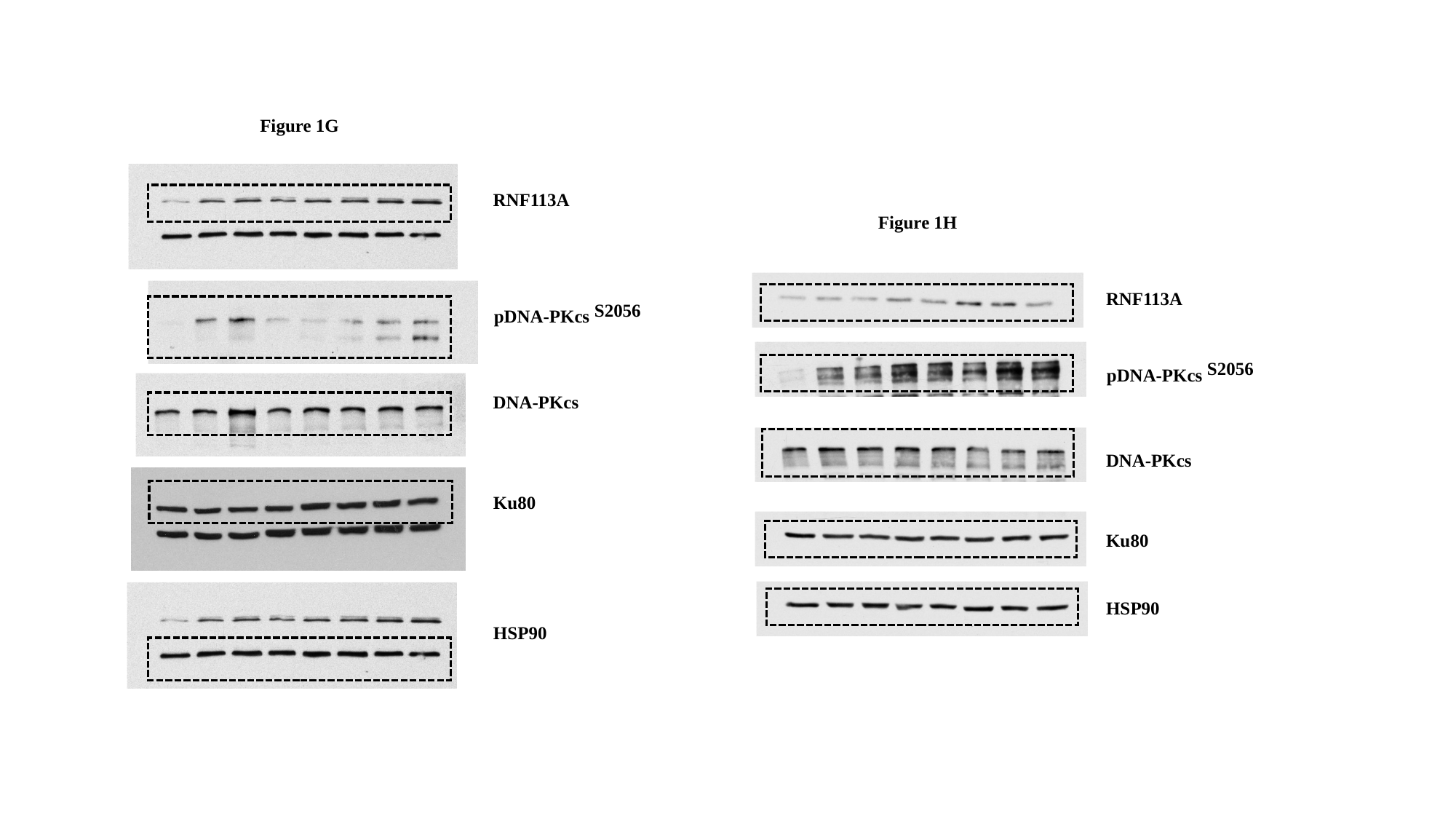

Figure 1G
RNF113A
Figure 1H
RNF113A
S2056
pDNA-PKcs
S2056
pDNA-PKcs
DNA-PKcs
DNA-PKcs
Ku80
Ku80
HSP90
HSP90

## Slide 3
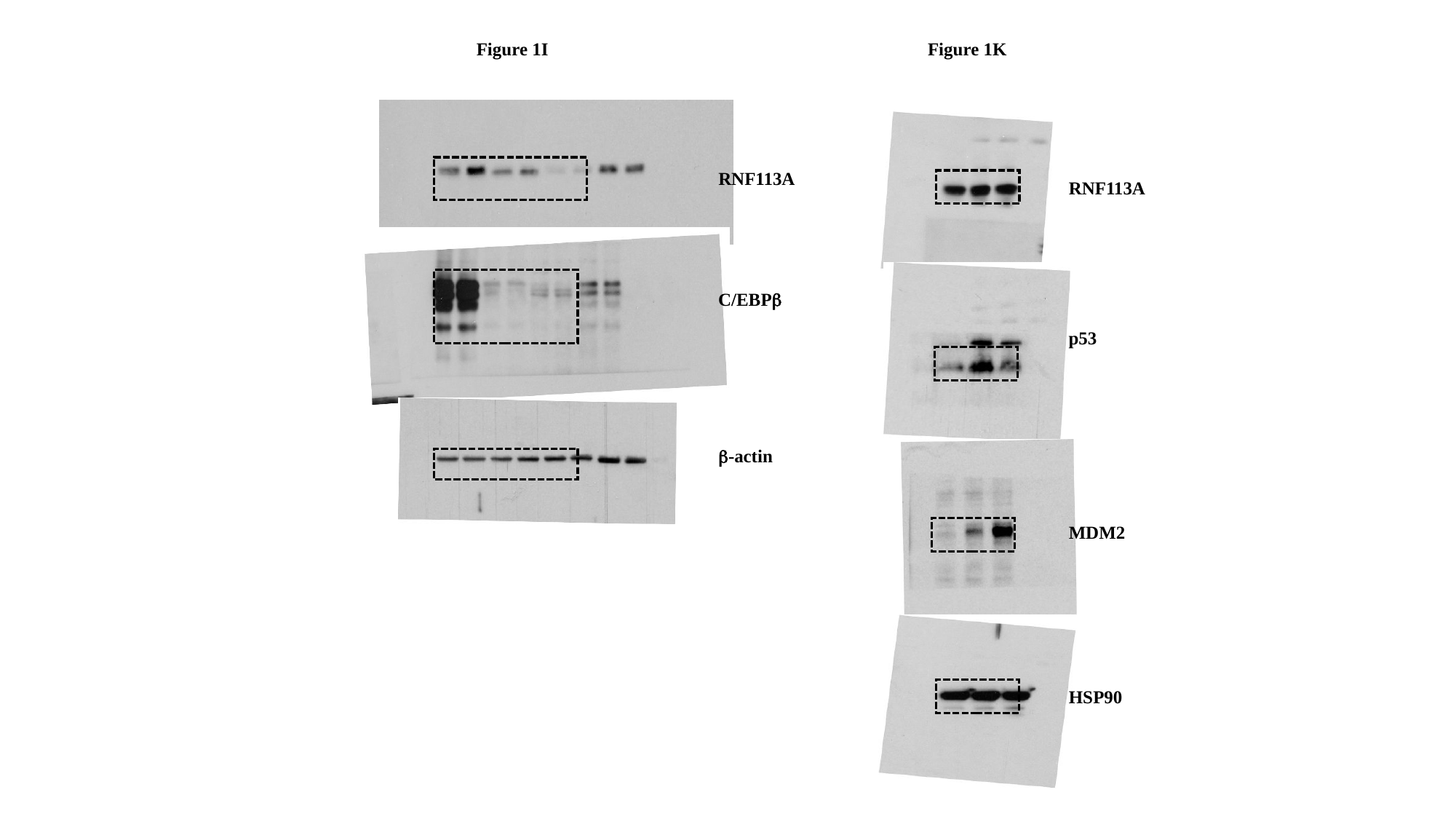

Figure 1I
Figure 1K
RNF113A
RNF113A
C/EBPb
p53
b-actin
MDM2
HSP90

## Slide 4
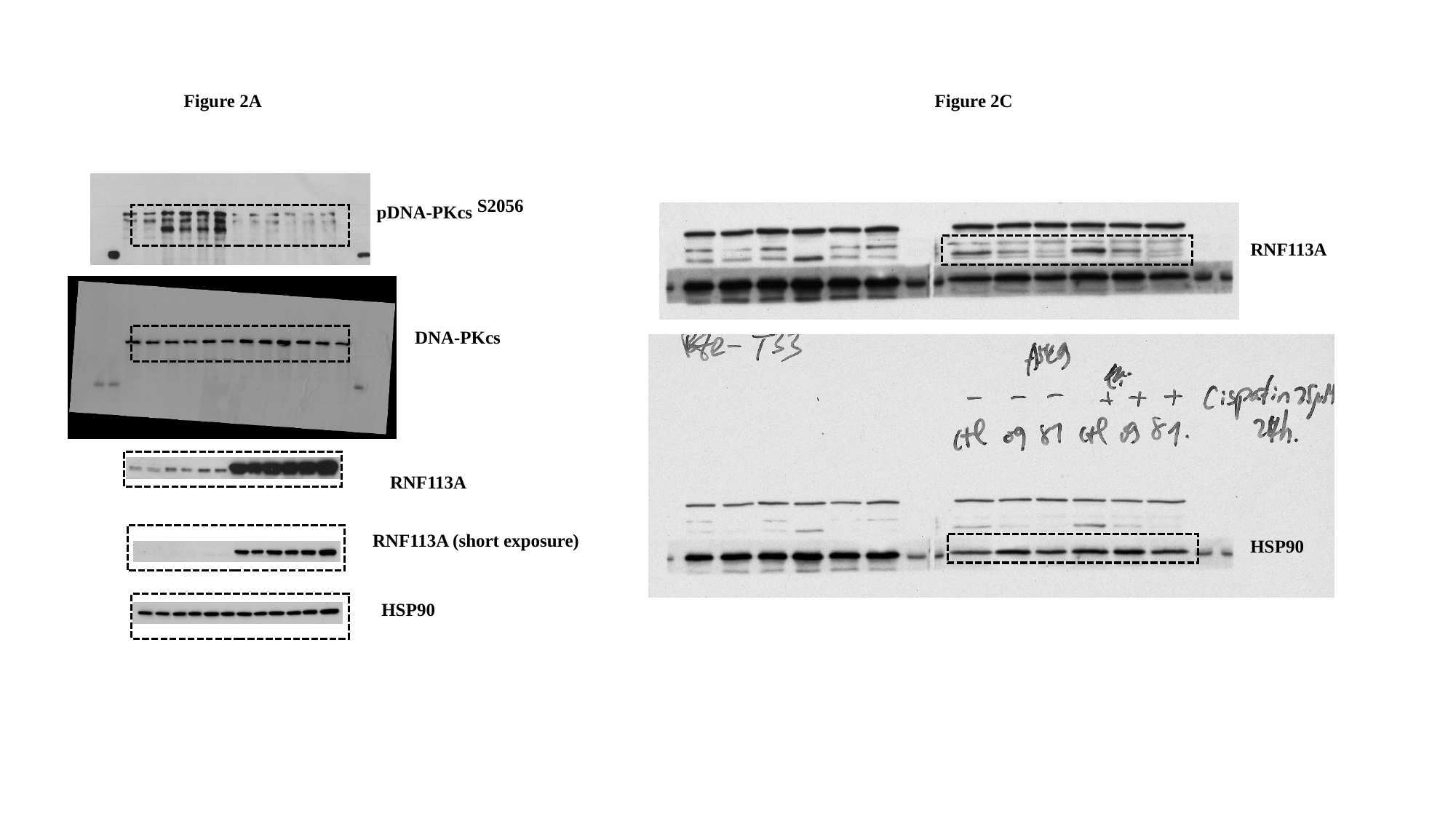

Figure 2C
Figure 2A
S2056
pDNA-PKcs
RNF113A
DNA-PKcs
RNF113A
RNF113A (short exposure)
HSP90
HSP90

## Slide 5
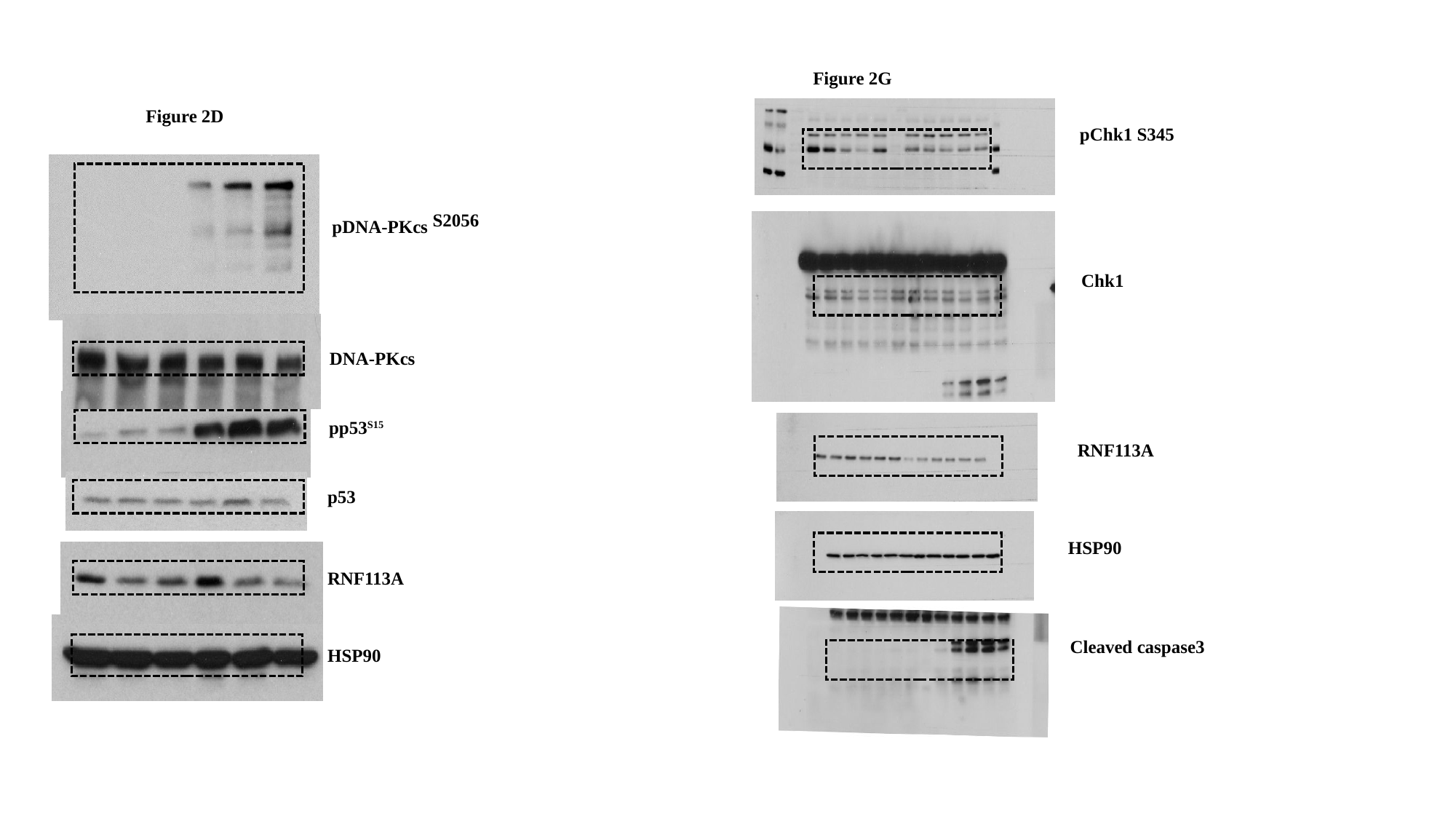

Figure 2G
Figure 2D
pChk1 S345
S2056
pDNA-PKcs
Chk1
DNA-PKcs
pp53S15
RNF113A
p53
HSP90
RNF113A
Cleaved caspase3
HSP90

## Slide 6
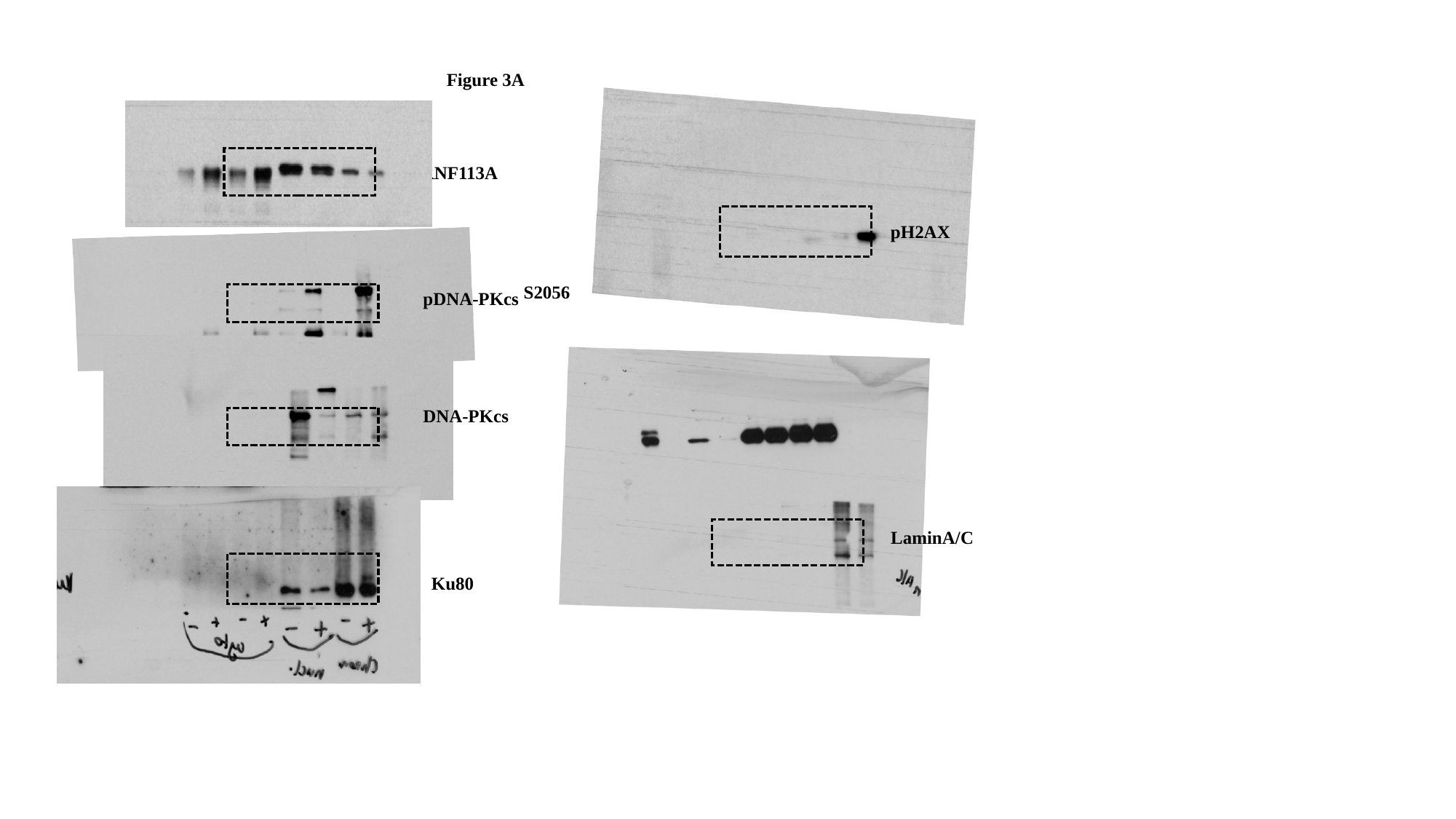

Figure 3A
RNF113A
pH2AX
S2056
pDNA-PKcs
DNA-PKcs
LaminA/C
Ku80

## Slide 7
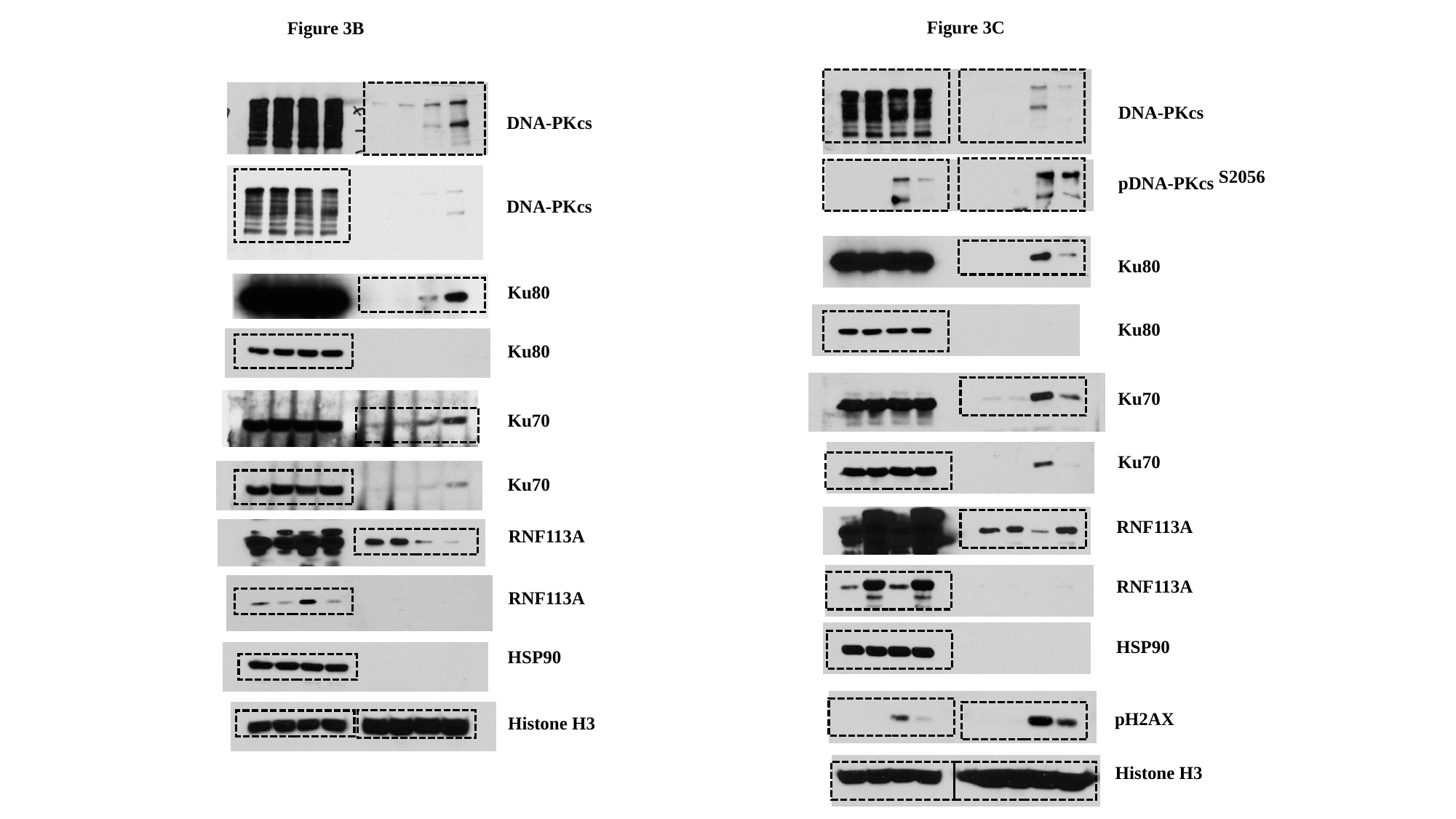

Figure 3C
Figure 3B
DNA-PKcs
DNA-PKcs
S2056
pDNA-PKcs
DNA-PKcs
Ku80
Ku80
Ku80
Ku80
Ku70
Ku70
Ku70
Ku70
RNF113A
RNF113A
RNF113A
RNF113A
HSP90
HSP90
pH2AX
Histone H3
Histone H3

## Slide 8
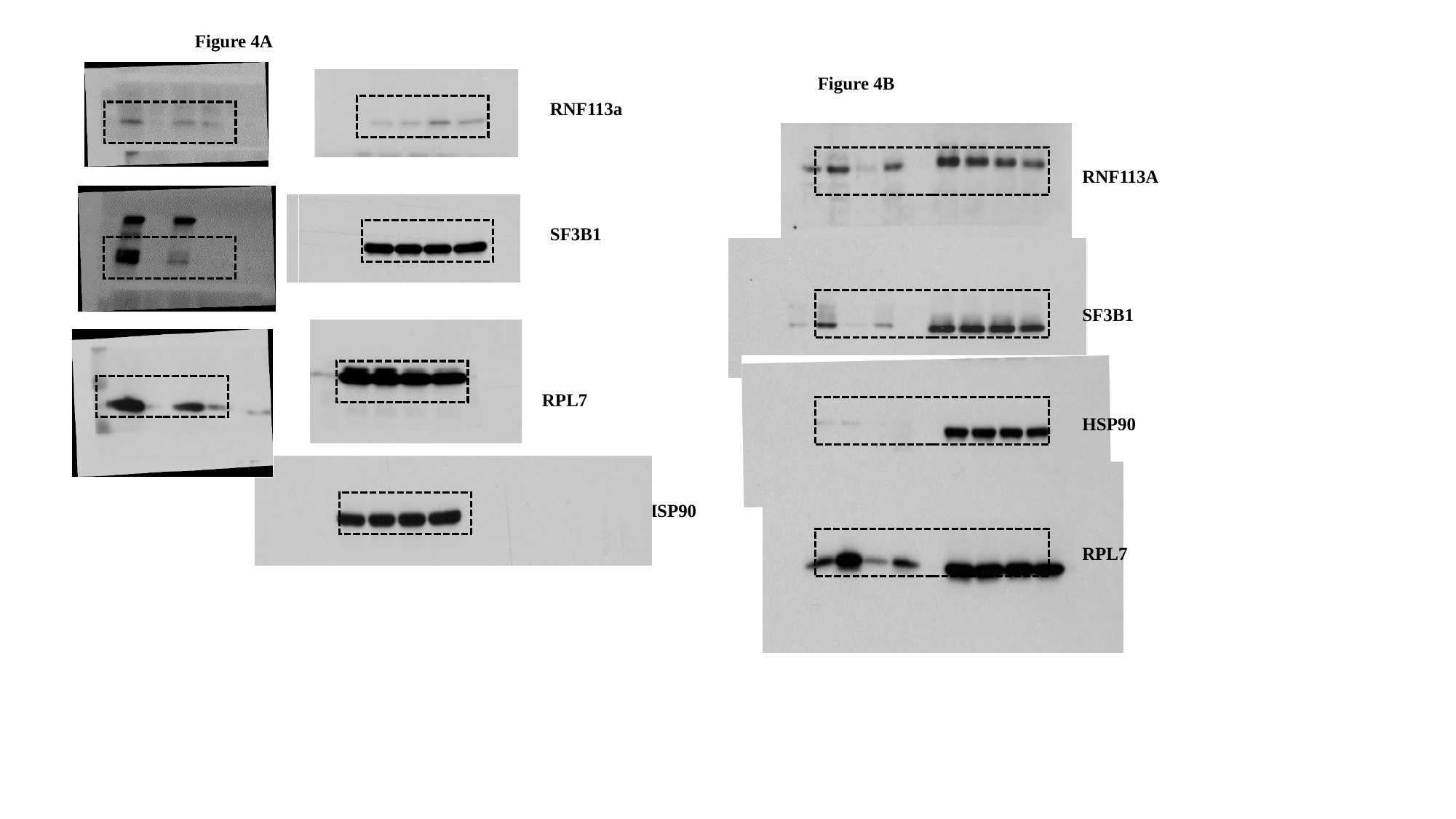

Figure 4A
Figure 4B
RNF113a
RNF113A
SF3B1
SF3B1
RPL7
HSP90
HSP90
RPL7

## Slide 9
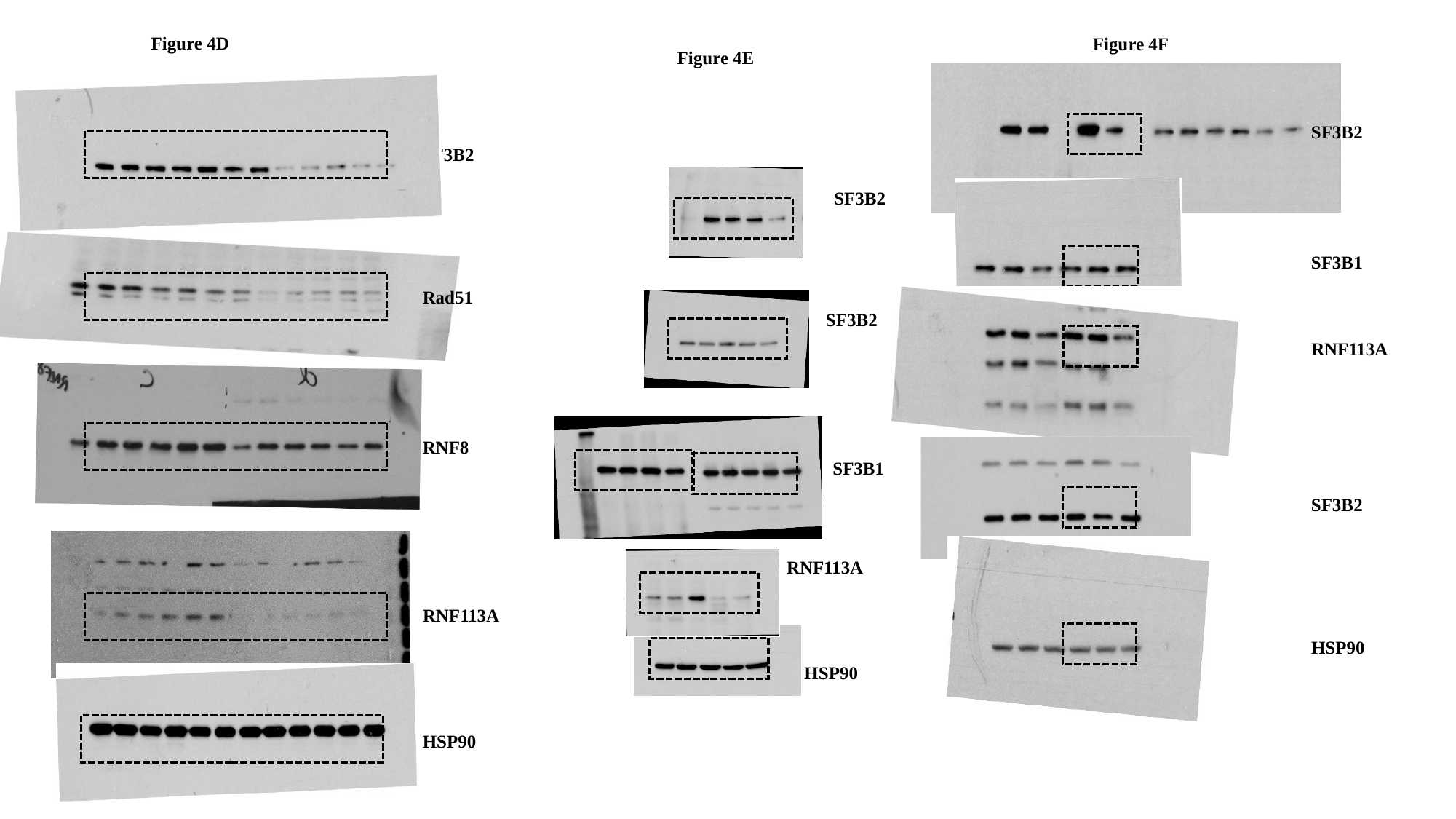

Figure 4D
Figure 4F
Figure 4E
SF3B2
SF3B2
SF3B2
SF3B1
Rad51
SF3B2
RNF113A
RNF8
SF3B1
SF3B2
RNF113A
RNF113A
HSP90
HSP90
HSP90

## Slide 10
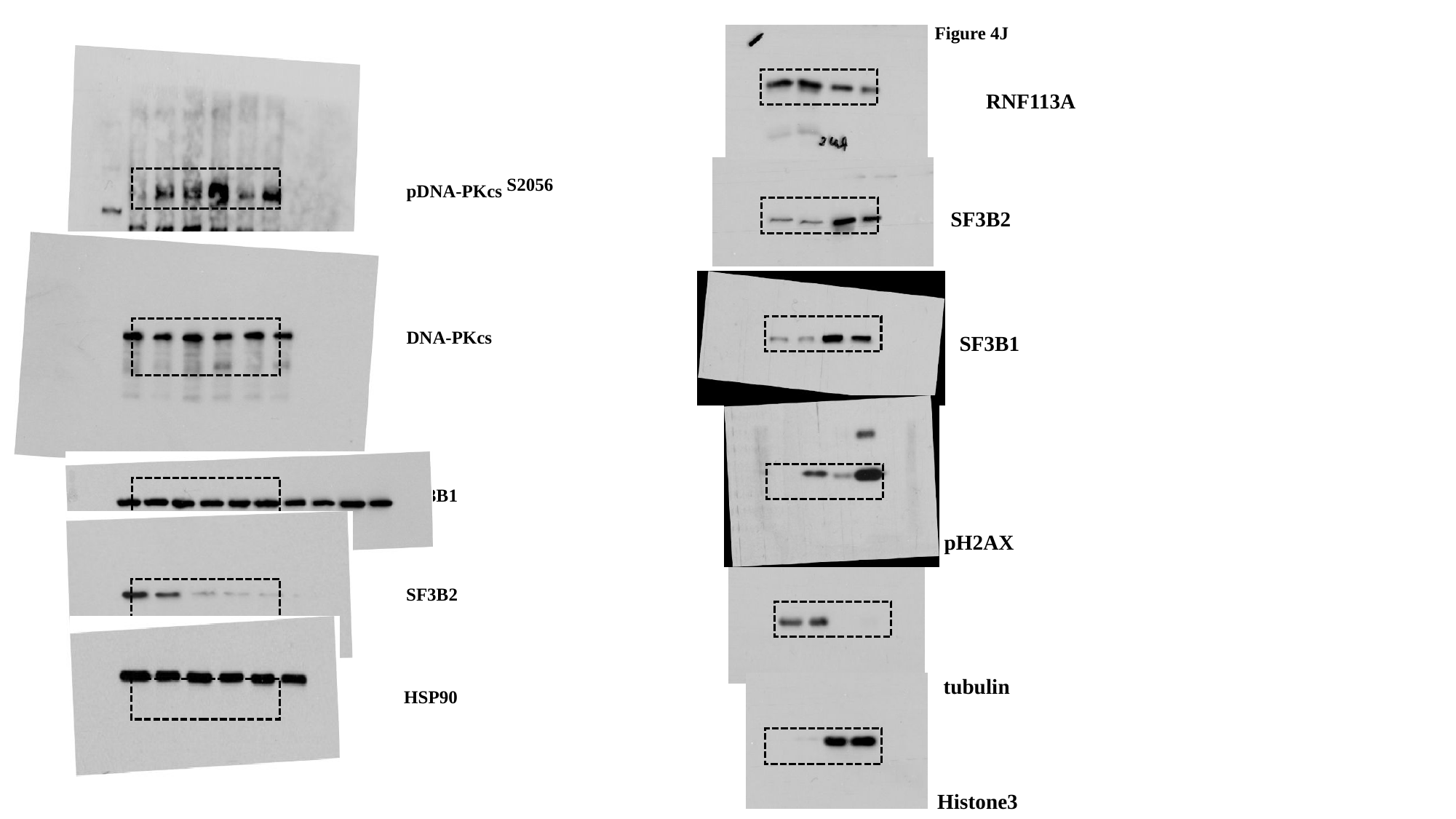

Figure 4J
Figure 4H
RNF113A
S2056
pDNA-PKcs
SF3B2
DNA-PKcs
SF3B1
SF3B1
pH2AX
SF3B2
tubulin
HSP90
Histone3

## Slide 11
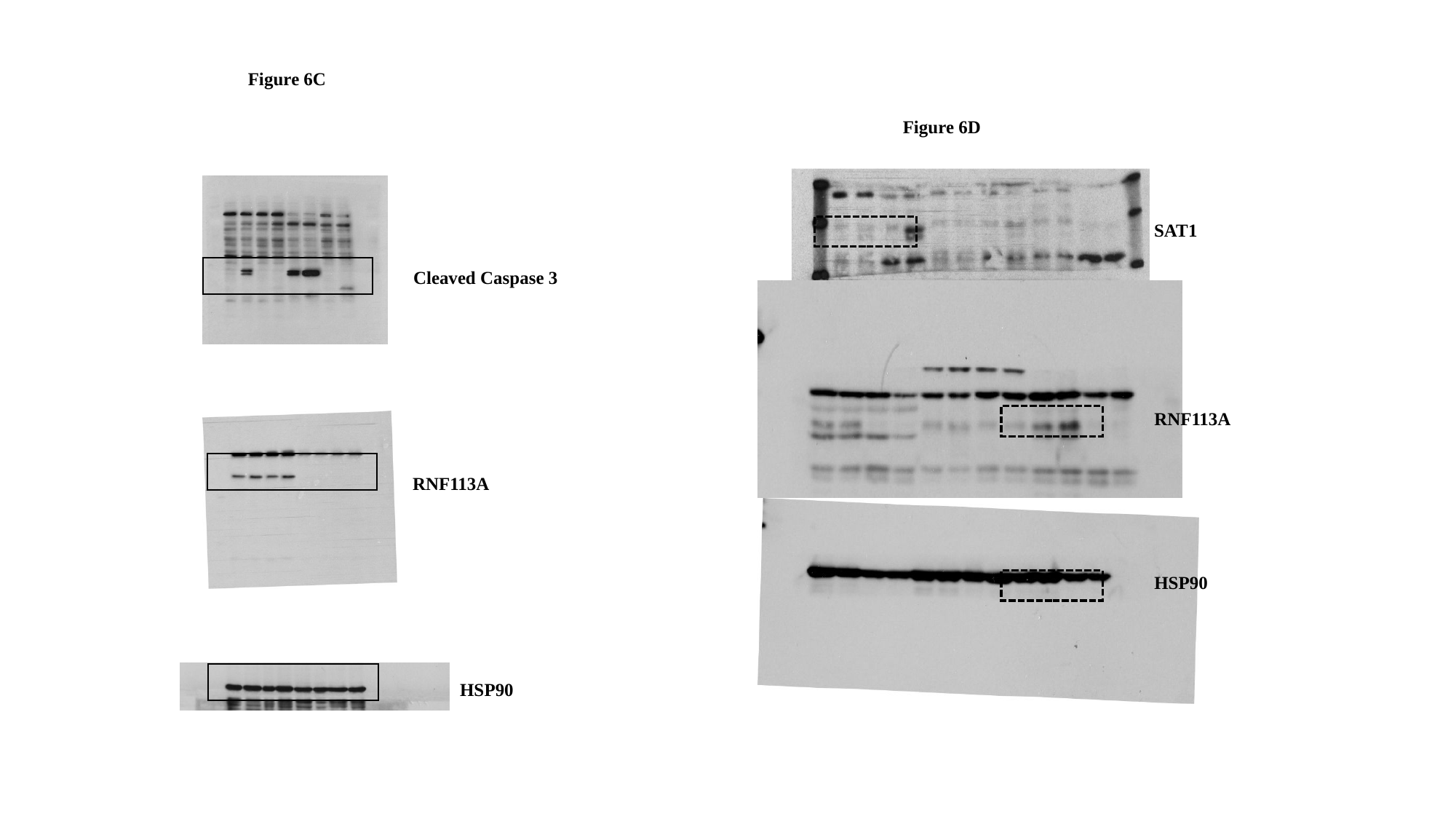

Figure 6C
Figure 6D
SAT1
Cleaved Caspase 3
RNF113A
RNF113A
HSP90
HSP90

## Slide 12
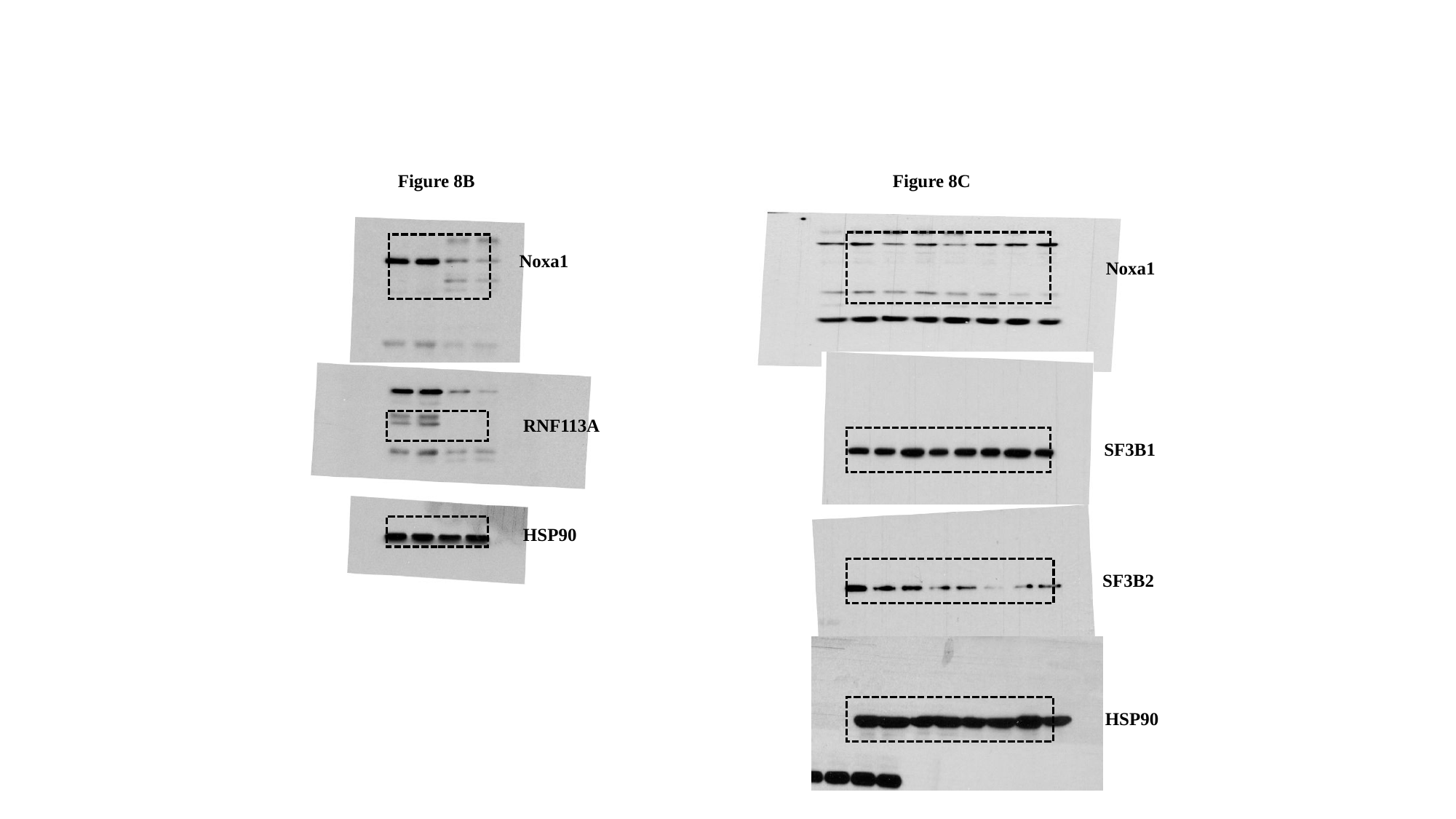

Figure 8B
Figure 8C
Noxa1
Noxa1
RNF113A
SF3B1
HSP90
SF3B2
HSP90

## Slide 13
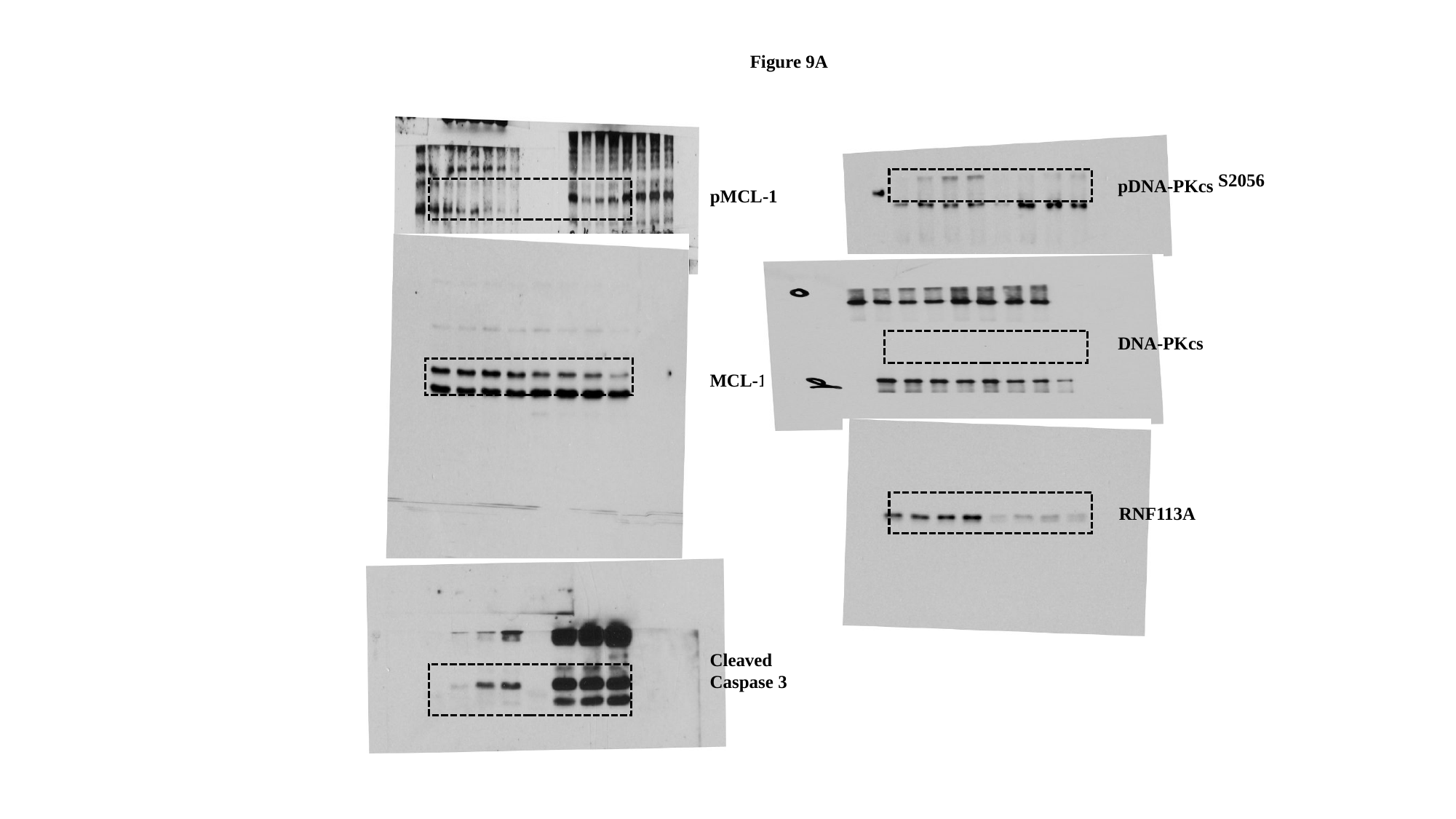

Figure 9A
S2056
pDNA-PKcs
pMCL-1
DNA-PKcs
MCL-1
RNF113A
Cleaved
Caspase 3

## Slide 14
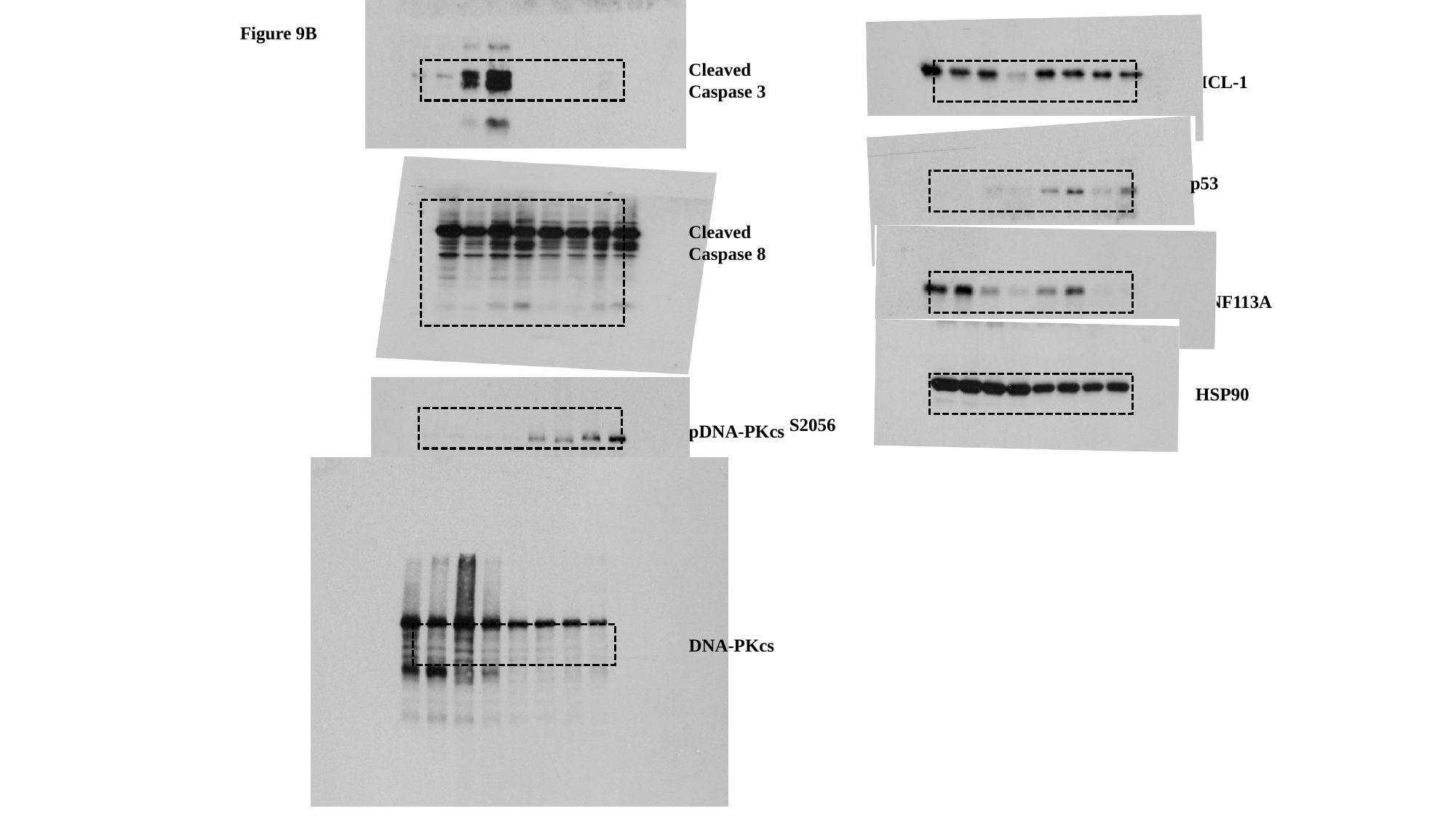

Figure 9B
Cleaved
Caspase 3
MCL-1
p53
Cleaved
Caspase 8
RNF113A
HSP90
S2056
pDNA-PKcs
DNA-PKcs

## Slide 15
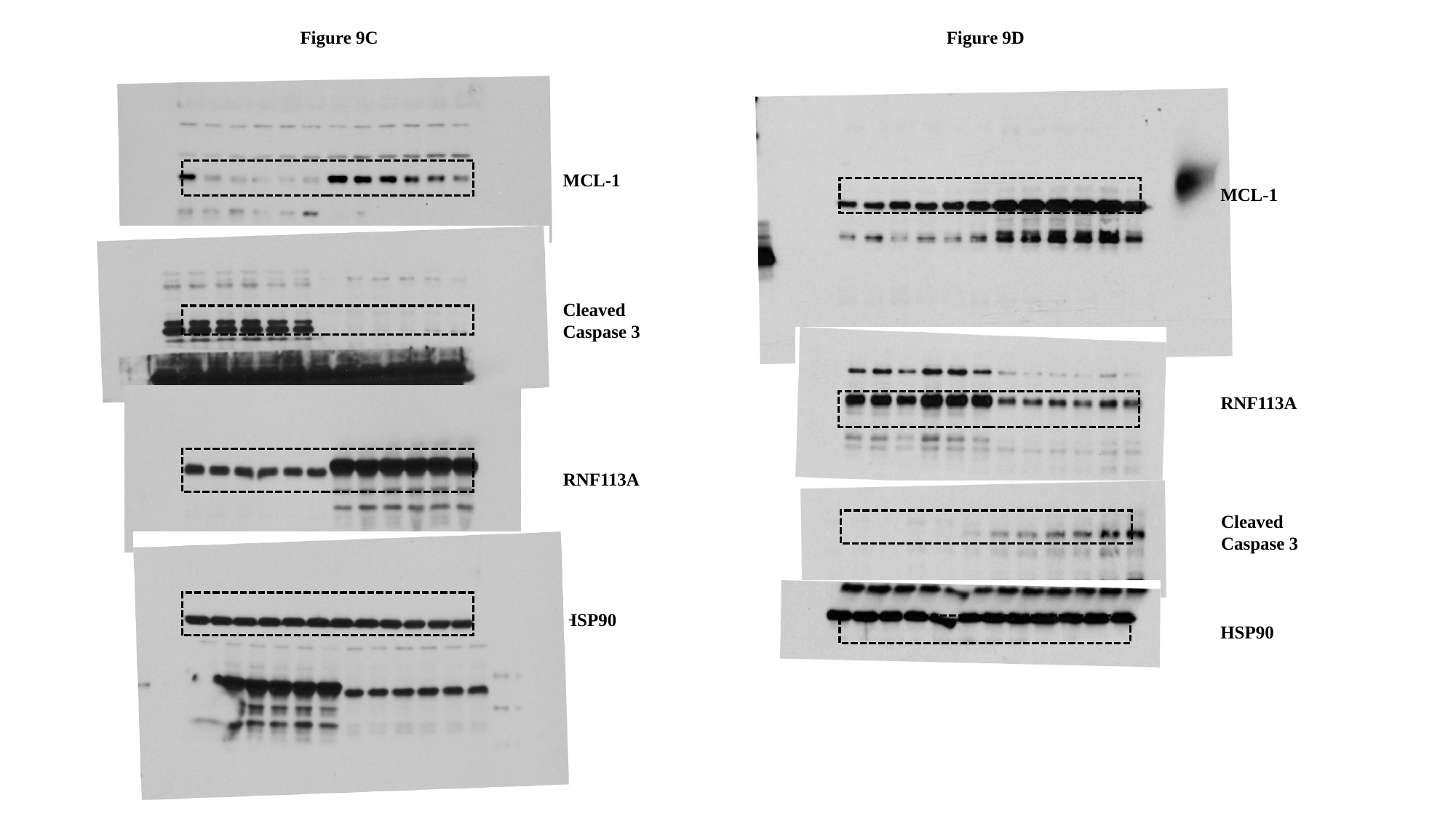

Figure 9C
Figure 9D
MCL-1
MCL-1
Cleaved
Caspase 3
RNF113A
RNF113A
Cleaved
Caspase 3
HSP90
HSP90

## Slide 16
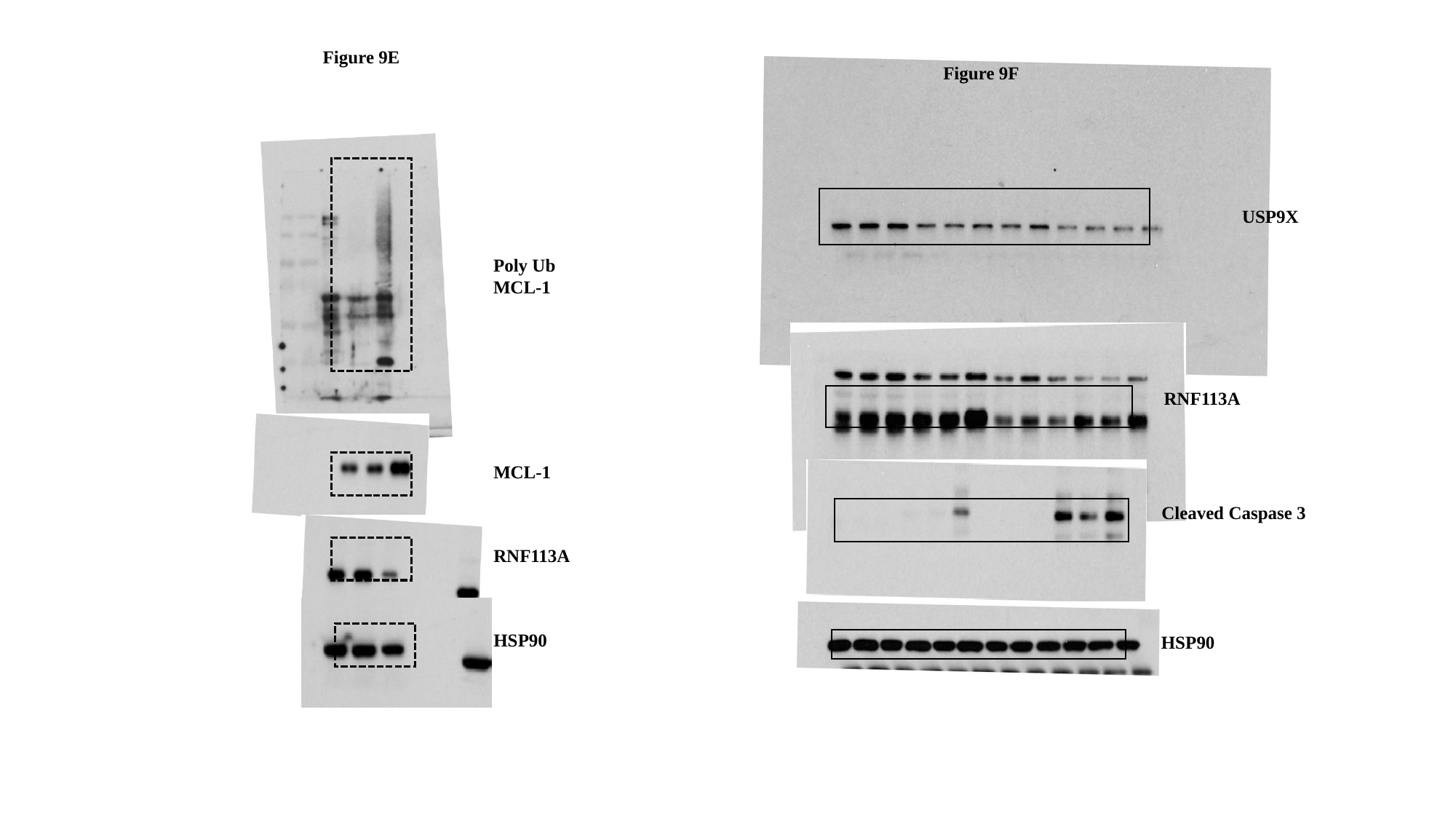

Figure 9E
Figure 9F
USP9X
Poly Ub
MCL-1
RNF113A
MCL-1
Cleaved Caspase 3
RNF113A
HSP90
HSP90

## Slide 17
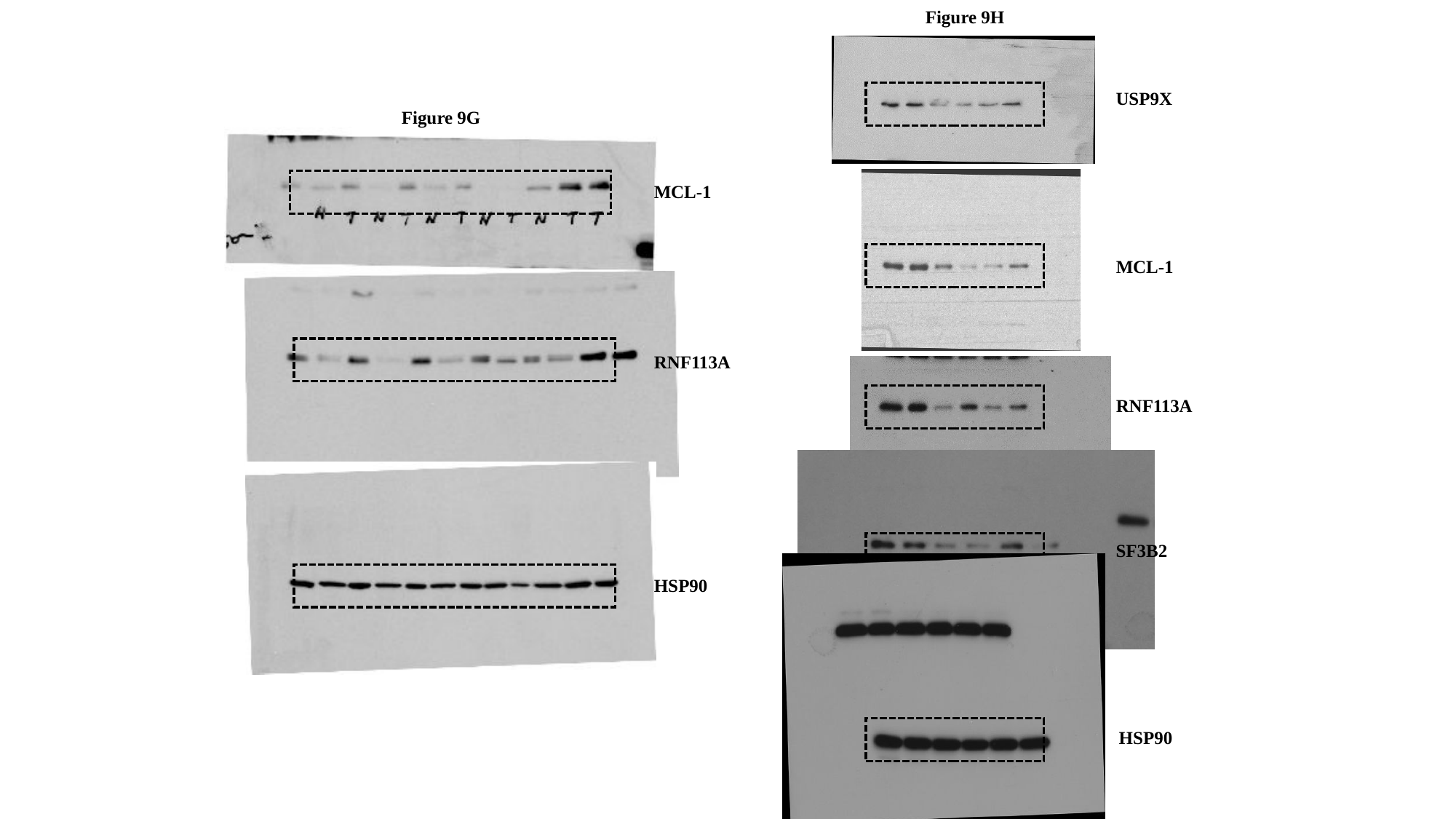

Figure 9H
USP9X
Figure 9G
MCL-1
MCL-1
RNF113A
RNF113A
SF3B2
HSP90
HSP90

## Slide 18
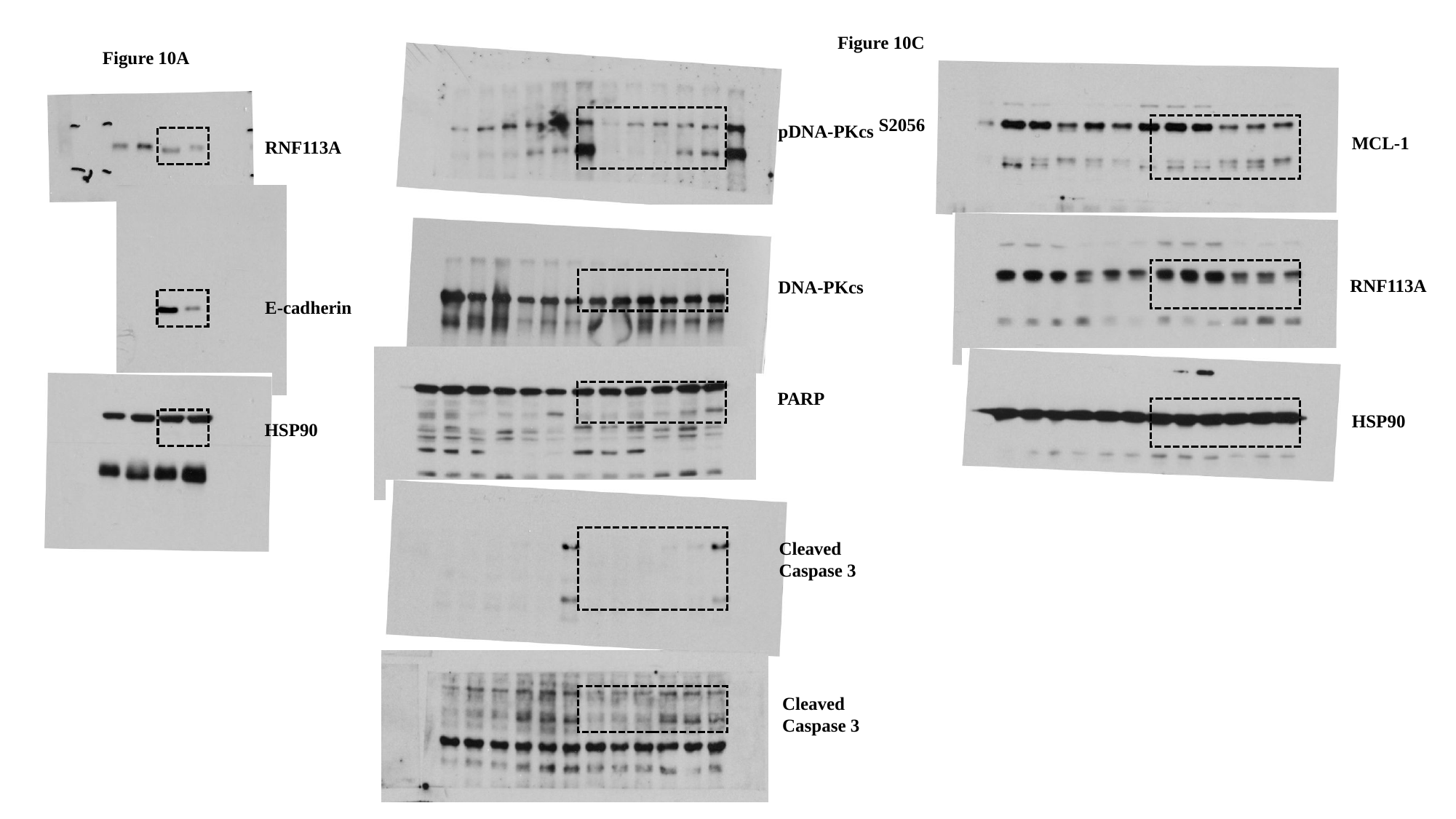

Figure 10C
Figure 10A
S2056
pDNA-PKcs
MCL-1
RNF113A
DNA-PKcs
RNF113A
E-cadherin
PARP
HSP90
HSP90
Cleaved
Caspase 3
Cleaved
Caspase 3

## Slide 19
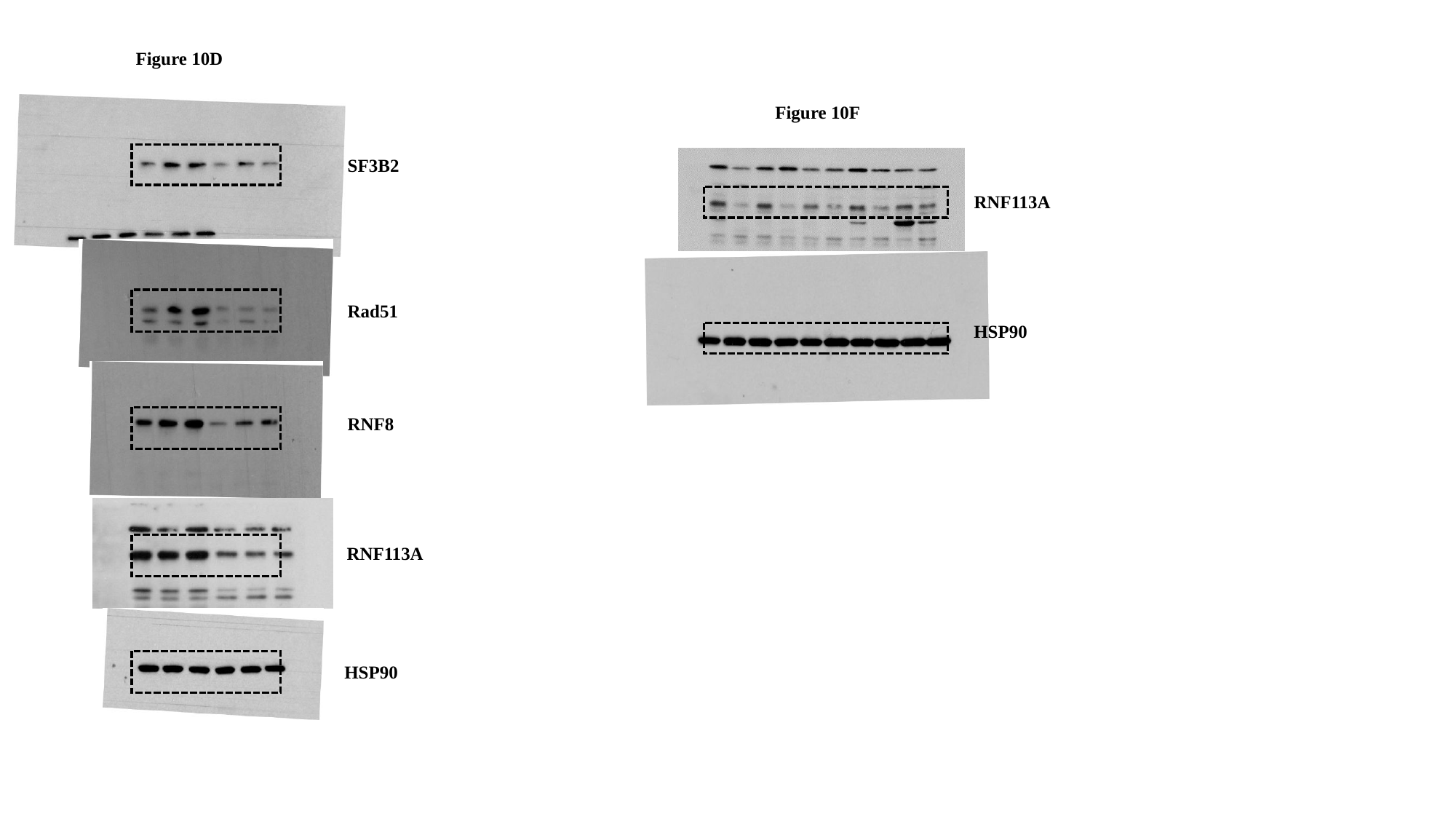

Figure 10D
Figure 10F
SF3B2
RNF113A
Rad51
HSP90
RNF8
RNF113A
HSP90
